# The congrid eel genus *Gnathophis* in Taiwan (Anguilliformes, Congridae), with descriptions of three new species

**DOI:** 10.3897/zookeys.1287.183842

**Published:** 2026-07-31

**Authors:** Jian-Fu Huang, Yun-Chih Liao, Tin-Yam Chan, Hong-Ming Chen, David G. Smith

**Affiliations:** 1 Institute of Marine Biology, National Taiwan Ocean University, Keelung 202, Taiwan Smithsonian Institution Washington United States of America; 2 Department of Earth and Life Science, University of Taipei, Taipei 100, Taiwan Department of Earth and Life Science, University of Taipei Taipei Taiwan; 3 Center of Excellence for the Oceans, National Taiwan Ocean University, Keelung 202, Taiwan Institute of Marine Biology, National Taiwan Ocean University Keelung Taiwan; 4 Department of Aquaculture, National Taiwan Ocean University, Keelung 202, Taiwan Center of Excellence for the Oceans, National Taiwan Ocean University Keelung Taiwan; 5 Smithsonian Institution, Washington, USA Department of Aquaculture, National Taiwan Ocean University Keelung Taiwan

**Keywords:** Biodiversity, Congrinae, DNA barcoding, new species, taxonomy

## Abstract

The genus *Gnathophis* differs from other genera of Congridae in having a moderately blunt and rounded tail tip and a slightly hardened tail extremity. The fleshy tip of the snout extends distinctly forwards beyond the intermaxillary teeth. A longitudinal fleshy keel is present on the ventral surface of the snout tip. Currently, two species of *Gnathophis* have been recorded from Taiwan, *G. heterognathos* and *G. asanoi*, whereas *G. xenica* has subsequently been determined to be a misidentification. In this study, three new species of the congrid eel genus *Gnathophis* Kaup are described from deep-sea waters off northeastern and southwestern Taiwan, based on 130 specimens. *Gnathophis kbalanensis***sp. nov**. is diagnosed by the combination of blackish dorsal, caudal, and anal fins; 34–36 preanal vertebrae, 42–43 precaudal vertebrae, and 134–137 total vertebrae; 32–35 preanal lateral line pores; and elevated lateral line pores above the pectoral fin. *Gnathophis melanurum***sp. nov**. is distinguished from its congeners by having whitish dorsal and anal fins and a blackish caudal fin; 28–32 preanal vertebrae, 37–41 precaudal vertebrae, and 98+–125 total vertebrae; 29–32 preanal lateral line pores; and elevated lateral line pores above the pectoral fin. *Gnathophis nanhaiensis***sp. nov**. is separable from other species of *Gnathophis* in possessing whitish dorsal, caudal, and anal fins; 40–42 preanal vertebrae, 47–48 precaudal vertebrae, and 139–143 total vertebrae; 37–41 preanal lateral line pores; and lateral line pores above the pectoral fin that are not elevated. The validities of *G. kbalanensis***sp. nov**. and *G. melanurum***sp. nov**. are further supported by DNA barcoding analysis of the mitochondrial cytochrome c oxidase subunit I (COI) gene. Molecular comparisons additionally indicate that specimens of *G. heterognathos* (Bleeker) from waters off Taiwan exhibit a maximum intraspecific COI sequence divergence of 1.4%.

This work is dedicated to the late Dr. David G. Smith for his enormous contribution to eel taxonomy and for greatly inspiring the first author of the present work.

## Introduction

The genus *Gnathophis* comprises medium-sized eels inhabiting tropical and subtropical deep waters worldwide. It is distinguished from other genera of the family Congridae by a combination of the following characters: tail moderately blunt, tip slightly stiffened, caudal fin short; snout projects beyond lower jaw, fleshy part of snout projects anteriorly well beyond intermaxillary teeth; longitudinal fleshy keel on underside of tip of snout (Smith 1989). Based on the condition of the lateral line pores above the pectoral fin, species of *Gnathophis* can be divided into two groups: those with elevated lateral line pores and those without elevated pores (Karmovskaya and Paxton 2000).

Kaup (1859) established the genus *Gnathophis* and included *Myrophis heterognathos* Bleeker, 1858 in the genus. The genus *Gnathophis* currently comprises 23 valid species distributed in the Indo-Pacific region (Matsubara and Ochiai 1951; Asano 1958; Kotthaus 1968; Karrer 1983; Karmovskaya 1990; Karmovskaya and Paxton 2000; Karmovskaya 2004; Kodeeswaran and Karmovskaya 2025; Prokofiev et al. 2025). In the northwestern Pacific, *G. heterognathos* (Bleeker, 1858) is known from Japan, Taiwan, and the South China Sea, including the Philippines (Karmovskaya 2004); *G. asanoi* Karmovskaya, 2004 has been recorded from the Philippines and Taiwan (Karmovskaya 2004; Ho et al. 2015, 2018); and *G. xenica* (Matsubara & Ochiai, 1951) and *G. ginanago* (Asanoi, 1958) are known only from Japan. In the southeastern Pacific, *G. andriashevi* Karmovskaya, 1990, *G. smithi* Karmovskaya, 1990, and *G. parini* Karmovskaya, 1990 are known from seamounts of the junction of the Nazca and Sala y Gómez ridges (Karmovskaya 1990). In the Indian Ocean, *G. heterolinea* (Kotthaus, 1968), *G. leptosomatus* Karrer, 1983 are distributed in the west Indian Ocean (Kotthaus 1968; Karrer 1983), and *G. ajithi* Kodeeswaran & Karmovskaya 2025, *G. anilmohapatrai* Kodeeswaran & Karmovskaya 2025, and *G. arabicus* Kodeeswaran & Karmovskaya 2025 are distributed in the Arabian Sea (Kodeeswaran and Karmovskaya 2025). In the western central Pacific Ocean, *G. johnsoni* (Prokofiev, Frable, Emelianova, Saveleva & Orlov, 2025) is known from seamounts of the Emperor–Hawaiian Chain (Prokofiev et al. 2025); *G. longicauda* (Ramsay & Ogilby, 1888) from Western Australia; *G. umbrellabius* (Whitley, 1948) from southeastern Australia and New Zealand; *G. macroporis* Karmovskaya & Paxton, 2000 from New South Wales and the coast of Victoria; *G. grahami* Karmovskaya & Paxton, 2000 and *G. castlei* Karmovskaya & Paxton, 2000 from eastern Australia; *G. microps* Karmovskaya & Paxton, 2000, *G. nasutus* Karmovskaya & Paxton, 2000, and *G. melanocoelus* Karmovskaya & Paxton, 2000 from northwestern Australia; *G. habenatus* (Richardson, 1848) from New Zealand (Karmovskaya and Paxton 2000); and *G. neocaledoniensis* Karmovskaya, 2004 from New Caledonia (Karmovskaya 2004).

*Gnathophis xenica* (Matsubara & Ochiai, 1951), previously listed in the Taiwan Fish Database, was later confirmed to be a misidentification. The four specimens deposited in ASIZP were identified as *Rhynchoconger ectenurus* (Jordan & Richardson, 1909) and *Bathycongrus* sp., whereas the two specimens deposited in FRIP were identified as *Congriscus maldivensis* (Norman, 1939) and the new species described in the present study. Although two species of *Gnathophis* have previously been recorded from Taiwan, the present study documents three additional, previously undescribed species from waters under the jurisdiction of Taiwan. One species was collected from the South China Sea, whereas the other two were obtained from waters off the coast of Taiwan main island.

## Materials and methods

Methods for measurements and counts follow Böhlke (1989). Vertebral counts were obtained from digital radiographs taken using X-ray equipment at the National Taiwan Ocean University and the National Museum of Marine Biology & Aquarium. Predorsal vertebrae were counted to a vertical through the first dorsal fin ray; preanal vertebrae to a vertical through the first anal fin ray; and precaudal vertebrae to the vertebra immediately anterior to the first centrum bearing a complete haemal spine. Counts of sensory pores were made under a light microscope. Predorsal pores were counted to a vertical just anterior to the first dorsal fin ray; prepectoral pores to a vertical through the upper base of the pectoral fin; preanal pores to a vertical through the posterior margin of the anus (or just anterior to the first anal fin ray); and total pores to the last pore on the tail.

Specimens were fixed in 10% formalin for one week and subsequently transferred to 70% ethanol for long-term preservation. Voucher specimens are deposited in the collections of the Laboratory of Aquatic Ecology, Department of Aquaculture, National Taiwan Ocean University, Keelung (**TOU-AE**); the Biodiversity Research Center, Academia Sinica, Taipei (**ASIZP**); the National Museum of Marine Biology & Aquarium, Pingtung (**NMMB-P**); and the Fisheries Research Institute, Keelung (**FRIP**).

### Abbreviations

**TL**, total length; **HL**, head length; **SO pores**, supraorbital pores; **IO pores**, infraorbital pores; **M pores**, mandibular pores; **POP pores**, preopercular pores; **ST pores**, supratemporal pores; **AN**, anterior nostril; **PN**, posterior nostril; **LL**, lateral line.

### Molecular methods

Muscle tissue samples were excised from selected fresh specimens and preserved in 95% ethanol, then stored at −20 °C prior to DNA extraction. Genomic DNA was extracted using a QIAGEN DNeasy Blood & Tissue Kit following the manufacturer’s protocols. A fragment of the mitochondrial cytochrome c oxidase subunit I (COI) gene was amplified by polymerase chain reaction (PCR) using the universal primers FishF1+2 (5’-TCR ACY AAY CAY AAA GAY ATY GGC AC-3’) and FishR2 (5’-ACT TCA GGG TGA CCG AAG AAT CAG AA-3’) (Chang et al. 2016).

PCR amplifications were performed in a total volume of 25 μL, containing 12.5 μL of 2X SuperRed PCR Master Mix, 3 μL of template DNA (50 ng μL^−1^), 7.5 μL of deionized water, and 1 μL for each primer. The thermal cycling profile consisted of an initial denaturation at 95 °C for 5 min, followed by 35 cycles of denaturation at 95 °C for 30 s, annealing at 50 °C for 30 s, and extension at 72 °C for 1 min, with a final extension at 72 °C for 8 min. PCR products were examined by agarose gel electrophoresis, purified, and sequenced by Genomics BioTech. Sequence chromatograms were assembled, trimmed, and aligned using Clustal W implemented in BioEdit v. 7.2.5 (Hall 1999), and final sequences were saved in FASTA format.

Newly generated COI sequences obtained in this study are listed in Table 1. An additional 20 COI sequences of *Gnathophis* spp. were retrieved from the National Center for Biotechnology Information (NCBI) GenBank database (accession numbers shown in figures) and included in phylogenetic analyses. A maximum-likelihood (ML) phylogenetic tree was reconstructed in MEGA v. X (Kumar et al. 2018) under the HKY+G nucleotide substitution model, selected as the best-fitting model based on the lowest bias-corrected Akaike Information Criterion (AICc) value. Node support was assessed using 1,000 bootstrap replicates (Felsenstein 1985). *Ariosoma fasciatum* (TOU-AE 9947; GenBank accession number PZ437811), subfamily Bathymyrinae, family Congridae, was designated as the outgroup. Intra- and interspecific genetic distances based on COI sequences were calculated using the Kimura two-parameter (K2P) model (Kimura 1980).

**Table 1.** Newly generated COI sequences of *Gnathophis* species in this study used for molecular analysis.

**Scientific name**	**Locality**	**Specimen number**	**Accession number**
*Gnathophis heterognathos*	Daxi, Yilan	TOU-AE 10869	PX648370
*Gnathophis heterognathos*	Daxi, Yilan	TOU-AE 10870	PX648371
*Gnathophis heterognathos*	Daxi, Yilan	TOU-AE 10871	PX648372
*Gnathophis heterognathos*	Badouzhi, Keelung	TOU-AE 9825	PX648359
*Gnathophis heterognathos*	Badouzhi, Keelung	TOU-AE 9826	PX648360
*Gnathophis heterognathos*	Badouzhi, Keelung	TOU-AE 9829	PX648361
*Gnathophis heterognathos*	Xianxi, Changhua	TOU-AE 8177	PX648362
*Gnathophis heterognathos*	Xianxi, Changhua	TOU-AE 8178	PX648363
*Gnathophis heterognathos*	Xianxi, Changhua	TOU-AE 8179	PX648364
*Gnathophis heterognathos*	Ke-tzu-liao, Kaohsiung	TOU-AE 11019	PX648353
*Gnathophis heterognathos*	Ke-tzu-liao, Kaohsiung	TOU-AE 11020	PX648354
*Gnathophis heterognathos*	Ke-tzu-liao, Kaohsiung	TOU-AE 11021	PX648355
*Gnathophis heterognathos*	Dong-gang, Pingtung	TOU-AE 11231	PX648349
*Gnathophis heterognathos*	Dong-gang, Pingtung	TOU-AE 11275	PX648348
*Gnathophis melanurum* sp. nov.	Daxi, Yilan	TOU-AE 8777	PX654100
*Gnathophis melanurum* sp. nov.	Daxi, Yilan	TOU-AE 8875	PX654103
*Gnathophis melanurum* sp. nov.	Daxi, Yilan	TOU-AE 8877	PX654104
*Gnathophis melanurum* sp. nov.	Badouzhi, Keelung	TOU-AE 9806	PX654105
*Gnathophis melanurum* sp. nov.	Dong-gang, Pingtung	TOU-AE 8006	PX654101
*Gnathophis melanurum* sp. nov.	Dong-gang, Pingtung	TOU-AE 8007	PX654102
*Gnathophis kbalanensis* sp. nov.	Daxi, Yilan	TOU-AE 9130	PX658967
*Gnathophis kbalanensis* sp. nov.	Daxi, Yilan	TOU-AE 9480	PX658966
*Gnathophis kbalanensis* sp. nov.	Daxi, Yilan	TOU-AE 10231	PX658968
*Gnathophis asanoi*	Dong-gang, Pingtung	NMMBP-30378	PX648477

## Taxonomic account

### Family Congridae

#### Genus Gnathophis

Taxon classificationAnimaliaAnguilliformesCongridae

Kaup, 1860

45A7F726-5471-5CB4-BE09-F76559D40C2B

Gnathophis Kaup, 1860: 7 (type species Myrophis heterognathos Bleeker, 1859, by original designation); Castle 1986: 164; Smith 1989: 521.
Rhynchocymba Jordan & Hubbs, 1925: 195 (type species Leptocephalus nystromi Jordan & Snyder, 1901, by original designation); Asano 1962: 92; Chen and Weng 1967: 51.

##### Diagnosis.

A genus of the subfamily Congrinae characterized by a stout to moderately elongate body. Trunk short, 16–26% TL. Preanal length 34–44% TL. Tail moderately attenuate, 55–65% TL. Dorsal fin origin slightly anterior to tip of appressed pectoral fin, continuous around tail tip with caudal and anal fins. Anal fin originating immediately posterior to anus. Pectoral fin well developed. Gill opening relatively small, smaller than eye diameter. Upper jaw projecting well beyond tip of lower jaw, distance between jaw tips ~1/3–1/2 of eye diameter. Eye of moderate size. Upper lip with a shallow, free, upturned flange; lower lip with a well-developed, downturned flange.

### Key to species of *Gnathophis* in Taiwan

**Table:** 

1	Lateral line pores above of pectoral fin are not elevated	**2**
–	Lateral line pores above of pectoral fin are elevated	**3**
2	Preanal vertebrae 34–37, precaudal vertebrae 42–44, preanal lateral line pores 35–37	***G. asanoi***
–	Preanal vertebrae 40–42, precaudal vertebrae 47–48, preanal lateral line pores 37–41	***G. nanhaiensis* sp. nov**.
3	Total vertebrae 134–137	***G. kbalanensis* sp. nov**.
–	Total vertebrae 120–127	**4**
4	Dorsal and anal fins pale in color without black margin, caudal fin black	***G. melanurum* sp. nov**.
–	Dorsal and anal fins with narrow black margin, caudal fin narrow and black	***G. heterognathos***

#### Gnathophis asanoi

Taxon classificationAnimaliaAnguilliformesCongridae

Karmovskaya, 2004

4BDE6F79-A100-5BE1-90DE-F7D72C75B0E3

**Figure 1.**
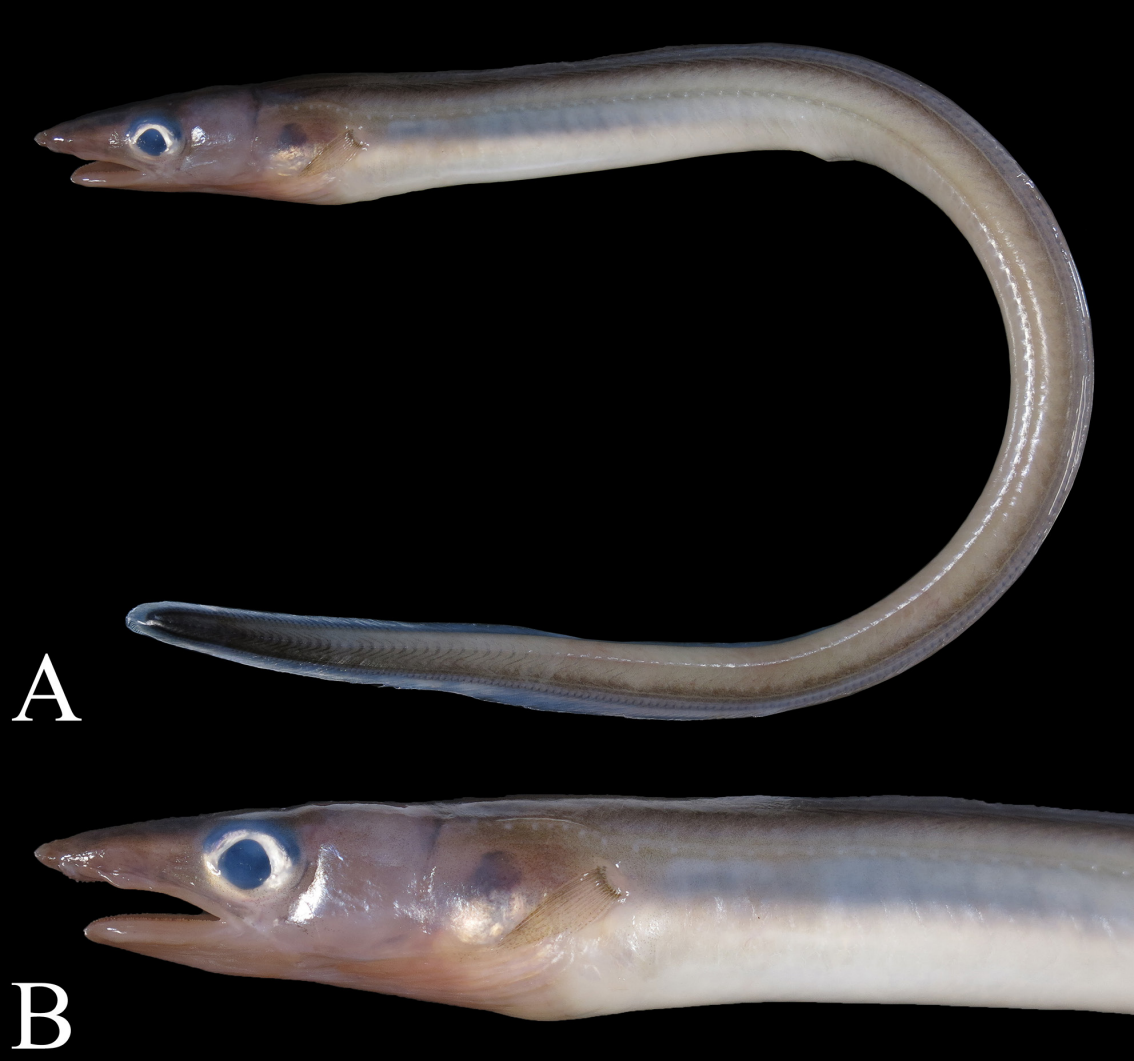
*Gnathophis asanoi* Karmovskaya, 2004, NMMB-P30378, 350 mm TL. **A**. Lateral view; **B**. Lateral view of anterior portion of head.

Figs 1, 6, 7; Tables 1, 2, 5, 7–11, 13

Gnathophis asanoi Karmovskaya, 2004: S18, fig. 10 (type locality: Philippines, Sibuyan Sea, 280–440 m); Ho et al. 2015: 147; Ho et al. 2018: 235, fig. 2.

##### English name.

Asano’s conger.

##### Chinese name.

淺野氏頜吻糯鰻.

##### Specimens examined.

• 3 specimens, 290–357 mm TL. • NMMB-P25987, 290 mm TL, Dong-gang, SW Taiwan, bottom trawl, 13 Oct. 2017, coll. H.-C. Ho; • NMMB-P30378, 350 mm TL, Dong-gang, SW Taiwan, bottom trawl, 01 Aug. 2018, coll. H.-C. Ho; • TOU-AE 6842, 357 mm TL, Dong-gang, SW Taiwan, bottom trawl, 04 Feb. 2013, coll. James Lin.

##### Diagnosis.

This species differs from all congeners of *Gnathophis* lacking elevated lateral line pores above the pectoral fin by the following combination of characters: dorsal portion of body near the dorsal fin base dark brown and distal portion of tail, beginning approximately one head length before the tail tip, gradually becoming dark; vertical fins and peritoneum pale; precaudal vertebrae 42–44; and predorsal length 16.0–16.9% TL.

##### Description of Taiwanese specimens.

Measurements and counts in Tables 5, 7. ***Body*** moderately elongate, cylindrical in cross section anteriorly, becoming laterally compressed posteriorly. Tail more than half of total length, ~ 0.6× TL, tip slightly rounded. Preanal length ~ 0.4 TL. Trunk length 0.3–0.4× tail length. Dorsal fin origin slightly anterior to tip of pectoral fin, continuous around tail tip with the caudal and anal fins. Predorsal length ~ 0.2 TL. Anal fin originating immediately posterior to anus. Pectoral fin well developed, distally pointed, with a narrow base. Gill opening relatively small, smaller than eye diameter, its upper margin nearly opposite the middle of the pectoral fin base; interbranchial width broader than gill opening and eye.

**Table 2.** Interspecific genetic distance of COI sequences of *Gnathophis* species calculated with Kimura-2-parameter model (Kimura 1980).

**COI**	**(1)**	**(2)**	**(3)**	**(4)**	**(5)**	**(6)**	**(7)**	**(8)**	**(9)**	**(10)**	**(11)**	**(12)**	**(13)**	**(14)**	**(15)**	**(16)**	**(17)**	**(18)**	**(19)**	**(20)**	**(21)**	**(22)**	**(23)**	**(24)**	**(25)**	**(26)**	**(27)**	**(28)**	**(29)**	**(30)**	**(31)**	**(32)**	**(33)**	**(34)**	**(35)**	**(36)**	**(37)**	**(38)**	**(39)**	**(40)**	**(41)**	**(42)**	**(43)**	**(44)**
(1) PX648348	–																																											
(2) PX648349	0.4%	–																																										
(3) PX648353	0.0%	0.4%	–																																									
(4) PX648354	0.0%	0.4%	0.0%	–																																								
(5) PX648355	1.0%	1.0%	1.0%	1.0%	–																																							
(6) PX648359	0.2%	0.6%	0.2%	0.2%	1.2%	–																																						
(7) PX648360	0.0%	0.4%	0.0%	0.0%	1.0%	0.2%	–																																					
(8) PX648361	0.4%	0.8%	0.4%	0.4%	1.4%	0.6%	0.4%	–																																				
(9) PX648362	0.2%	0.6%	0.2%	0.2%	1.2%	0.4%	0.2%	0.6%	–																																			
(10) PX648363	0.4%	0.8%	0.4%	0.4%	1.4%	0.6%	0.4%	0.8%	0.6%	–																																		
(11) PX648364	0.2%	0.6%	0.2%	0.2%	1.2%	0.4%	0.2%	0.6%	0.4%	0.6%	–																																	
(12) PX648370	0.2%	0.6%	0.2%	0.2%	1.2%	0.4%	0.2%	0.6%	0.4%	0.6%	0.4%	–																																
(13) PX648371	0.4%	0.8%	0.4%	0.4%	1.4%	0.6%	0.4%	0.8%	0.6%	0.8%	0.6%	0.6%	–																															
(14) PX648372	0.0%	0.4%	0.0%	0.0%	1.0%	0.2%	0.0%	0.4%	0.2%	0.4%	0.2%	0.2%	0.4%	–																														
(15) KF681849	1.2%	1.6%	1.2%	1.2%	1.4%	1.4%	1.2%	1.6%	1.4%	1.6%	1.4%	1.4%	1.6%	1.2%	–																													
(16) KP267595	1.4%	1.8%	1.4%	1.4%	1.2%	1.6%	1.4%	1.8%	1.6%	1.8%	1.6%	1.6%	1.8%	1.4%	1.8%	–																												
(17) PX654100	5.3%	5.3%	5.3%	5.3%	5.9%	5.5%	5.3%	5.7%	5.5%	5.3%	5.1%	5.5%	5.7%	5.3%	6.1%	5.7%	–																											
(18) PX654101	5.3%	5.3%	5.3%	5.3%	5.9%	5.5%	5.3%	5.7%	5.5%	5.3%	5.1%	5.5%	5.7%	5.3%	6.1%	5.7%	0.0%	–																										
(19) PX654102	5.7%	5.7%	5.7%	5.7%	6.4%	5.9%	5.7%	5.7%	5.9%	5.7%	5.5%	5.9%	6.2%	5.7%	6.6%	6.1%	0.4%	0.4%	–																									
(20) PX654103	5.3%	5.3%	5.3%	5.3%	5.9%	5.5%	5.3%	5.7%	5.5%	5.3%	5.1%	5.5%	5.7%	5.3%	6.1%	5.7%	0.0%	0.0%	0.4%	–																								
(21) PX654104	5.3%	5.3%	5.3%	5.3%	5.9%	5.5%	5.3%	5.7%	5.5%	5.3%	5.1%	5.5%	5.7%	5.3%	6.1%	5.7%	0.0%	0.0%	0.4%	0.0%	–																							
(22) PX654105	5.5%	5.5%	5.5%	5.5%	6.2%	5.7%	5.5%	5.9%	5.7%	5.5%	5.3%	5.7%	5.9%	5.5%	6.4%	5.9%	0.2%	0.2%	0.6%	0.2%	0.2%	–																						
(23) PX658966	4.9%	4.9%	4.9%	4.9%	5.5%	4.7%	4.9%	5.3%	5.1%	5.3%	5.1%	5.1%	4.9%	4.9%	5.7%	5.5%	4.4%	4.4%	4.9%	4.4%	4.4%	4.7%	–																					
(24) PX658967	4.9%	4.9%	4.9%	4.9%	5.5%	4.7%	4.9%	5.3%	5.1%	5.3%	5.1%	5.1%	4.9%	4.9%	5.7%	5.5%	4.4%	4.4%	4.9%	4.4%	4.4%	4.7%	0.0%	–																				
(25) PX658968	4.9%	4.9%	4.9%	4.9%	5.5%	4.7%	4.9%	5.3%	5.1%	5.3%	5.1%	5.1%	4.9%	4.9%	5.7%	5.5%	4.4%	4.4%	4.9%	4.4%	4.4%	4.7%	0.0%	0.0%	–																			
(26) PX648477	15.7%	15.7%	15.7%	15.7%	16.2%	15.5%	15.7%	16.2%	16.0%	15.7%	15.5%	16.0%	15.2%	15.7%	16.4%	15.7%	12.7%	12.7%	12.7%	12.7%	12.7%	12.5%	12.7%	12.7%	12.7%	–																		
(27) PV455848	5.1%	5.1%	5.1%	5.1%	5.7%	5.3%	5.1%	5.5%	5.3%	5.1%	5.3%	5.3%	5.5%	5.1%	5.9%	5.5%	2.2%	2.2%	2.6%	2.2%	2.2%	2.4%	4.2%	4.2%	4.2%	13.5%	–																	
(28) PV455847	4.9%	4.9%	4.9%	4.9%	5.5%	5.1%	4.9%	5.3%	5.1%	4.9%	5.1%	5.1%	5.3%	4.9%	5.7%	5.3%	2.0%	2.0%	2.4%	2.0%	2.0%	2.2%	4.0%	4.0%	4.0%	13.5%	0.2%	–																
(29) HM902538	3.2%	3.6%	3.2%	3.2%	3.4%	3.4%	3.2%	3.6%	3.4%	3.4%	3.4%	3.4%	3.6%	3.2%	3.6%	3.8%	6.1%	6.1%	6.6%	6.1%	6.1%	6.4%	5.7%	5.7%	5.7%	16.5%	6.1%	5.9%	–															
(30) HM902537	3.0%	3.4%	3.0%	3.0%	3.2%	3.2%	3.0%	3.4%	3.2%	3.2%	3.2%	3.2%	3.4%	3.0%	3.4%	3.2%	5.9%	5.9%	6.4%	5.9%	5.9%	6.1%	5.9%	5.9%	5.9%	16.5%	5.9%	5.7%	0.8%	–														
(31) HM902535	10.4%	10.4%	10.4%	10.4%	11.1%	10.6%	10.4%	9.9%	10.1%	10.1%	10.6%	10.6%	10.4%	10.4%	10.3%	10.8%	8.8%	8.8%	8.3%	8.8%	8.8%	9.0%	9.2%	9.2%	9.2%	14.0%	9.2%	9.0%	11.3%	11.1%	–													
(32) HM902532	10.1%	10.1%	10.1%	10.1%	10.8%	10.4%	10.1%	9.7%	9.9%	9.9%	10.4%	10.4%	10.1%	10.1%	10.1%	10.6%	8.1%	8.1%	7.6%	8.1%	8.1%	8.3%	8.5%	8.5%	8.5%	13.3%	8.5%	8.3%	11.1%	10.8%	0.6%	–												
(33) HM902533	7.4%	7.4%	7.4%	7.4%	7.6%	7.6%	7.4%	7.8%	7.6%	7.6%	7.6%	7.6%	7.4%	7.4%	8.3%	7.8%	8.1%	8.1%	8.5%	8.1%	8.1%	8.3%	6.3%	6.3%	6.3%	13.9%	7.4%	7.2%	8.3%	8.5%	10.6%	9.9%	–											
(34) HM902540	6.7%	6.7%	6.7%	6.7%	7.0%	7.0%	6.7%	7.2%	7.0%	7.0%	7.0%	7.0%	6.7%	6.7%	7.6%	7.2%	7.4%	7.4%	7.9%	7.4%	7.4%	7.6%	6.1%	6.1%	6.1%	13.4%	7.2%	7.0%	7.8%	8.1%	10.1%	9.4%	0.6%	–										
(35) JF493549	7.6%	7.6%	7.6%	7.6%	7.4%	7.9%	7.6%	8.1%	7.9%	7.9%	7.9%	7.9%	8.1%	7.6%	8.1%	7.6%	6.8%	6.8%	7.2%	6.8%	6.8%	7.0%	5.3%	5.3%	5.3%	13.9%	6.6%	6.3%	8.3%	8.5%	10.6%	9.9%	4.4%	4.2%	–									
(36) JF493548	7.9%	7.9%	7.9%	7.9%	8.1%	8.1%	7.9%	8.3%	8.1%	8.1%	8.1%	8.1%	7.9%	7.9%	8.7%	8.3%	7.4%	7.4%	7.9%	7.4%	7.4%	7.7%	5.5%	5.5%	5.5%	14.2%	7.2%	7.0%	8.5%	8.8%	10.4%	9.7%	3.8%	3.6%	1.0%	–								
(37) KC015423	4.7%	4.7%	4.7%	4.7%	5.7%	4.9%	4.7%	5.1%	4.9%	5.1%	4.9%	4.9%	4.7%	4.7%	5.9%	5.7%	4.7%	4.7%	5.1%	4.7%	4.7%	4.9%	1.0%	1.0%	1.0%	13.7%	4.0%	3.8%	5.5%	5.7%	9.4%	8.8%	6.6%	6.3%	5.5%	5.7%	–							
(38) KC015422	5.9%	5.9%	5.9%	5.9%	6.6%	6.1%	5.9%	6.4%	6.1%	6.4%	6.1%	6.1%	5.9%	5.9%	6.8%	6.6%	5.5%	5.5%	5.9%	5.5%	5.5%	5.7%	1.8%	1.8%	1.8%	14.9%	4.9%	4.6%	6.3%	6.5%	9.9%	9.6%	6.8%	6.8%	6.1%	6.3%	1.2%	–						
(39) MN022249	5.9%	6.1%	5.9%	5.9%	6.1%	6.1%	5.9%	6.4%	6.1%	5.5%	5.7%	6.1%	6.4%	5.9%	6.8%	6.3%	2.0%	2.0%	2.4%	2.0%	2.0%	2.2%	5.3%	5.3%	5.3%	13.9%	2.2%	2.0%	6.8%	6.6%	9.6%	9.0%	7.4%	7.2%	6.5%	7.2%	5.1%	5.9%	–					
(40) PV839149	2.4%	2.8%	2.4%	2.4%	3.0%	2.6%	2.4%	2.8%	2.6%	2.6%	2.6%	2.6%	2.8%	2.4%	3.6%	3.4%	6.4%	6.4%	6.8%	6.4%	6.4%	6.6%	5.5%	5.5%	5.5%	16.2%	6.6%	6.4%	3.2%	3.0%	12.0%	11.8%	8.1%	7.4%	8.1%	8.4%	5.3%	6.6%	7.2%	–				
(41) PV839150	2.4%	2.8%	2.4%	2.4%	3.0%	2.6%	2.4%	2.8%	2.6%	2.6%	2.6%	2.6%	2.8%	2.4%	3.6%	3.4%	5.9%	5.9%	6.4%	5.9%	5.9%	6.1%	5.1%	5.1%	5.1%	15.7%	6.1%	5.9%	3.2%	3.0%	12.0%	11.3%	7.7%	7.0%	7.7%	7.9%	4.9%	6.1%	6.8%	0.4%	–			
(42) PV839151	15.2%	15.2%	15.2%	15.2%	15.7%	15.4%	15.2%	15.7%	15.4%	15.2%	14.9%	14.9%	15.2%	15.2%	15.1%	14.4%	12.5%	12.5%	12.9%	12.5%	12.5%	12.7%	13.7%	13.7%	13.7%	12.8%	13.4%	13.2%	15.2%	15.1%	15.8%	15.5%	14.6%	14.4%	14.4%	14.7%	14.2%	14.5%	13.2%	14.6%	14.1%	–		
(43) PV839152	13.4%	13.9%	13.4%	13.4%	14.2%	13.7%	13.4%	13.9%	13.7%	13.4%	13.2%	13.7%	13.4%	13.4%	14.2%	13.4%	12.2%	12.2%	12.7%	12.2%	12.2%	12.0%	12.9%	12.9%	12.9%	10.6%	12.2%	12.0%	14.7%	14.1%	14.8%	14.0%	14.1%	13.6%	15.2%	15.9%	13.7%	14.1%	13.1%	14.1%	13.6%	13.5%	–	
(44) PV839153	13.2%	13.7%	13.2%	13.2%	14.4%	13.4%	13.2%	13.7%	13.4%	13.2%	12.9%	13.4%	13.2%	13.2%	14.4%	13.6%	12.5%	12.5%	12.9%	12.5%	12.5%	12.2%	13.2%	13.2%	13.2%	10.6%	12.5%	12.2%	14.9%	14.4%	15.0%	14.3%	14.4%	13.9%	15.4%	16.2%	13.4%	14.4%	13.4%	13.9%	13.4%	13.5%	0.2%	–

***Head*** moderately large, reflecting less slender body, deepest at gill opening. Head length 0.6–0.7× trunk length and ~ 0.4× preanal length. Snout long and pointed, 1.6–1.8× eye diameter, projecting well beyond lower jaw; lower jaw distinctly shorter than snout. Fleshy portion of snout projecting anteriorly beyond anterior margin of intermaxillary tooth patch. Rictus located below anterior half of eye. Anterior nostril tubular, near tip of snout, directed ventrolaterally. Posterior nostril elliptical, situated anterior to eye at mid-eye level. Upper lip with a shallow, free, upturned flange, beginning at second infraorbital pore and ending below middle of eye. Lower lip with a well-developed, downturned flange.

***Lateral line*** complete; first pore on each side slightly enlarged; canal extending to caudal fin base; 6–7 before pectoral-fin base, 9–10 before dorsal fin origin, and 35–37 before anal fin origin; lateral line pores above pectoral fin not elevated.

***Head pores*** small (Fig. 6A). Supraorbital canal with five pores: first (ethmoidal pore) and second on ventral surface of snout tip just anterior to upper lip; third on dorsal surface of snout tip; fourth on dorsal surface of anterior half of snout; fifth above posterior margin of eye. Infraorbital canal with seven pores, first four along upper lip, fifth posterior to rictus, and two pores behind eye. Frontal pore absent. Preopercular canal with two pores. Mandibular canal with seven pores, six along lower jaw and the last well posterior to rictus. Supratemporal commissure with three pores, the median pore minute. Predorsal vertebrae 9–11; preanal vertebrae 34–37; precaudal vertebrae 42–44; total vertebrae 139–141.

***Teeth*** small, conical to blunt (Fig. 7C). Intermaxillary teeth arranged in 4–5 transverse rows, continuous with maxillary and vomerine tooth patches. Maxillary and mandibular teeth in bands, broader anteriorly with ~ 4–5 rows, narrowing posteriorly to ~ 2–3 rows. Vomerine teeth blunt, forming an elongate patch of ~ 3–4 transverse rows.

##### Coloration.

When fresh, dorsal surface of head and dorsal portion of body near the dorsal fin base dark brown. Lateral surfaces of head and upper and lower lips brown; ventral surface of head light brown. Lateral line pores and the lateral to ventral surfaces of body pale white to grayish white. Distal portion of tail, beginning approximately one head length before the tail tip, gradually becoming dark. Vertical fins pale. Pectoral fin pale, with sparse pigmentation. Peritoneum pale.

##### Size.

Maximum observed total length 357 mm.

##### Distribution.

Known from the northwestern part of the Sibuyan Sea, Philippines, extending to waters off southwestern Taiwan, at depths of ~ 200–400 m.

##### Remarks.

*Gnathophis asanoi* belongs to the species group characterized by lateral line pores above the pectoral fin not elevated. The species was originally described from the Philippines based on two type specimens (Karmovskaya 2004). Ho et al. (2018) subsequently recorded this species from southwestern Taiwan based on two specimens.

#### Gnathophis heterognathos

Taxon classificationAnimaliaAnguilliformesCongridae

(Bleeker, 1858)

81C55429-66B1-57D3-900E-9063A17AB52E

**Figure 2.**
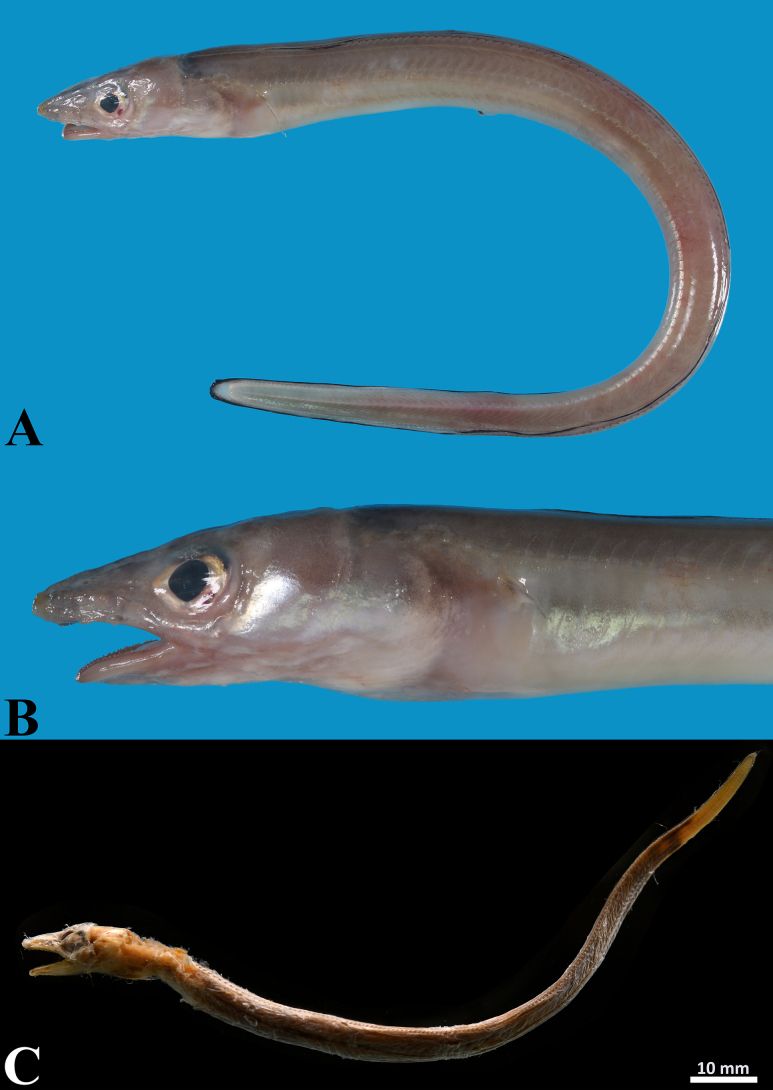
*Gnathophis heterognathos* (Bleeker, 1858), TOU-AE 9900, 329 mm TL. **A**. Lateral view; **B**. Lateral view of anterior portion of head; **C**. Holotype of *Myrophis heterognathos* Bleeker, 1858, BMNH 1867.11.28.305, 137 mm TL.

Figs 2, 6, 8; Tables 1, 2, 3, 6, 8–12

Myrophis heterognathos Bleeker, 1858: 9, pl. 3, fig. 1 (type locality: Nagasaki, Japan).
Gnathophis heterognathos (Bleeker, 1858): Kaup 1859: 7; Castle 1963: 17; Karmovskaya 2004: S15, fig. 8; Ho et al. 2015: 8.
Leptocephalus nystromi Jordan & Snyder, 1901: 853, fig. 5 (type locality: Nagasaki, Japan).
Ariosoma nystromi nystromi (Jordan & Snyder, 1901): Matsubara and Ochiai 1951: 5, fig. 5.
Rhynchocymba nystromi (Jordan & Snyder, 1901): Chen and Weng 1967: 51; Chen 1969: 132; Shen 1984a: 112; Chen and Yu 1986: 252.
Rhynchocymba nystromi nystromi (Jordan & Snyder, 1901): Chu 1957: 14; Asano 1962: 93, fig. 40; Liang and Yeh 1964: 8; Lee and Yang 1966: 57, fig. 6; Shao et al. 2008: 239.
Gnathophis nystromi (Jordan & Snyder, 1901): Shen 1984b: 14, pl. 14; Shen 1988: 15; Shao et al. 1994: 274; Chen 2004: 32.
Rhynchocymba ntstromi ginanago Asano, 1958: 7, fig. 1; Asano 1962: 96, fig. 41 (type locality: Japan).
Gnathophis nystromi ginanago (Asano, 1958): Shao et al. 1994: 274.
Gnathophis nystromi nystromi (Jordan & Snyder, 1901): Shen and Wu 2011: 139.

##### English name.

Black tail conger.

##### Chinese name.

異頜吻糯鰻.

##### Specimens examined.

• 74 specimens, 155+–483 mm TL. • **Daxi, Yilan, northeastern Taiwan**. TOU-AE 8775, 244 mm TL, TOU-AE 8776, 252 mm TL, 27 Aug. 2022. • TOU-AE 9900, 329 mm TL, 08 Jun. 2023. TOU-AE 9696, 230 mm TL, TOU-AE 9699, 260 mm TL, a ripe female and free eggs, TOU-AE 9702, 214 mm TL, a ripe female and free eggs, 24 Oct. 2022. • TOU-AE 10868, 201+ mm TL, TOU-AE 10869, 173 mm TL, TOU-AE 10870, 203 mm TL, TOU-AE 10871, 196 mm TL, 07 Jan. 2025. TOU-AE 8031, 251 mm TL, 05 Feb. 2021. NMMB-P29902, 308 mm TL, 05 Dec. 2014. • NMMB-P11903, 371 mm TL, 21 Jan. 2011. • **Badouzhi, Keelung, northeastern Taiwan**. TOU-AE 9814, 450 mm TL, TOU-AE 9815, 401 mm TL, TOU-AE 9816, 419 mm TL, TOU-AE 9817, 460 mm TL, TOU-AE 9818, 426 mm TL, TOU-AE 9819, 440 mm TL, TOU-AE 9820, 414 mm TL, TOU-AE 9821, 459 mm TL, TOU-AE 9822, 449 mm TL, TOU-AE 9823, 446 mm TL, TOU-AE 9824, 447 mm TL, TOU-AE 9825, 378 mm TL, TOU-AE 9826, 340 mm TL, TOU-AE 9827, 377 mm TL, TOU-AE 9828, 370 mm TL, TOU-AE 9829, 401 mm TL, TOU-AE 9830, 375 mm TL, TOU-AE 9831, 273 mm TL, TOU-AE 9832, 483 mm TL, TOU-AE 9833, 313 mm TL, TOU-AE 9834, 376 mm TL, TOU-AE 9835, 345 mm TL, TOU-AE 9836, 463 mm TL, TOU-AE 9837, 355 mm TL, TOU-AE 9841, 400 mm TL, TOU-AE 9845, 246 mm TL, TOU-AE 9846, 299 mm TL, TOU-AE 9848, 286 mm TL, TOU-AE 9852, 358 mm TL, TOU-AE 9855, 379 mm TL, 15 May. 2023. • **Xianxi, Changhua, southwestern Taiwan**. TOU-AE 8177, 223+ mm TL, 05 Feb. 2021. TOU-AE 8178, 231 mm TL, TOU-AE 8180, 234 mm TL, 01 Feb. 2021. TOU-AE 8179, 290 mm TL, 07 Feb. 2021. TOU-AE 8181, 295 mm TL, 25 Feb. 2021. • TOU-AE 8021, 180 mm TL, 27 Jan. 2021. • TOU-AE 8022, 267 mm TL, 27 Feb. 2021. • **Ke-tzu-liao, Kaohsiung, southwestern Taiwan**. TOU-AE 10165, 309 mm TL, TOU-AE 10166, 195+ mm TL, TOU-AE 10167, 170 mm TL, TOU-AE 10168, 169 mm TL, TOU-AE 10169, 160+ mm TL, TOU-AE 10170, 168 mm TL, TOU-AE 10171, 177 mm TL, 07 Jan. 2024. • TOU-AE 10139, 175 mm TL, 18 Sep. 2023. • TOU-AE 11015, 288 mm TL, TOU-AE 11016, 311 mm TL, TOU-AE 11017, 253 mm TL, TOU-AE 11018, 209 mm TL, TOU-AE 11019, 223 mm TL, TOU-AE 11020, 216 mm TL, TOU-AE 11021, 228 mm TL, 05 Nov. 2023. TOU-AE 10644, 155+ mm TL, TOU-AE 10645, 221 mm TL, TOU-AE 10646, 175 mm TL, 07 Nov. 2023. • NMMB-P25291, 235 mm TL, 31 Oct. 2016. • NMMB-P22740, 172 mm TL, 11 Feb. 2015. • NMMB-P28590, 200 mm TL, 08 Feb. 2018. • **Dong-gang, Pingtung, southwestern, Taiwan**. NMMB-P31510, 164 mm TL, 04 Sep. 2017. • TOU-AE 11275, 188 mm TL, 23 Oct. 2025. • TOU-AE 11231, 164 mm TL, 31 May. 2025.

##### Diagnosis.

This species can be distinguished from all other congeners possessing elevated lateral line pores above the pectoral fin by the following combination of characters: a pale peritoneum; snout length 26.4–32.1% HL; precaudal vertebrae 33–42; total vertebrae 120–127; and POP pores 3.

##### Description of Taiwanese specimens.

Measurements and counts in Tables 3, 6. ***Body*** moderately elongate, cylindrical in cross section anteriorly, becoming laterally compressed posteriorly. Tail 0.6–0.7× TL, tip slightly rounded. Preanal length 0.3–0.4 TL. Trunk length 0.3–0.5× tail length. Dorsal-fin origin slightly posterior to base of pectoral fin, continuous around tip of tail with caudal and anal fins. Predorsal length ~ 0.2 TL. Anal fin originating immediately posterior to the anus. Pectoral fin well developed, distally pointed, with a narrow base. Gill opening relatively small, smaller than eye diameter, its upper margin nearly opposite the middle of pectoral-fin base; interbranchial width broader than gill opening and eye.

**Table 3.** Morphometric data of *Gnathophis heterognathos* and *Myrophis heterognathos*.

	***Gnathophis heterognathos***	***Myrophis heterognathos***
		**BMNH 1867.11.28.305**
Total length (mm)	155+–483 (*n* = 70)	137.3
As % of TL	Mean (Range)	
Head length	17.8 (16.2–19.6)	17.0
Predorsal length	19.5 (17.3–21.0)	20.7
Preanal length	39.2 (34.1–44.2)	38.4
Trunk length	21.3 (16.6–26.2)	21.4
Tail length	60.8 (55.8–65.9)	–
Depth at gill opening	6.2 (4.7–7.6)	–
Depth at anus	5.3 (4.2–6.9)	–
As % of PAL		
Head length	45.8 (40.8–51.4)	–
Trunk length	54.2 (48.6–59.2)	–
Predorsal length	49.9 (45.4–57.0)	–
Depth at gill opening	15.7 (12.2–19.2)	–
Depth at anus	13.5 (10.9–17.0)	–
As % of HL		
Snout length	29.3 (26.4–32.1)	27.6
Eye diameter	19.7 (15.8–26.2)	17.0
Interorbital width	7.9 (4.8–15.5)	–
Interbranchial width	23.0 (16.6–32.7)	–
Gill opening width	11.6 (6.8–15.6)	–
Upper jaw length	37.3 (31.6–41.9)	–
Lower jaw length	29.0 (24.1–32.7)	–
Pectoral-fin length	30.8 (23.0–38.0)	27.7

***Head*** moderately large, reflecting less slender body, deepest at gill opening. Head length 0.7–1.1× trunk length and 0.4–0.5× preanal length. Snout long and pointed, 1.1–1.9× eye diameter, projecting well beyond lower jaw; lower jaw distinctly shorter than snout. Fleshy portion of snout projecting anteriorly beyond the anterior margin of intermaxillary tooth patch. Rictus located below anterior half of the eye. Anterior nostril tubular, near tip of snout, directed ventrolaterally. Posterior nostril elliptical, situated anterior to eye at mid-eye level. Upper lip with a shallow, free, upturned flange, beginning at the second infraorbital pore and ending below middle of the eye. Lower lip with a well-developed, downturned flange.

***Lateral line*** complete; first pore on each side slightly enlarged; canal extending to base of caudal fin. 5–8 pores before pectoral fin base, 7–11 before dorsal fin origin, and 22–35 before anal fin origin. Lateral line pores above pectoral fin elevated, extending approximately from pores 6–9 anteriorly to pores 11–15 posteriorly; second pore also slightly elevated.

***Head pores*** small (Fig. 6B). Supraorbital canal with six pores: first (ethmoidal pore) and second on ventral surface of snout tip just anterior to upper lip; third on dorsal surface of snout tip; fourth on dorsal surface of anterior half of snout; fifth above anterior margin of eye; sixth above posterior margin of eye. Infraorbital canal with eight pores, first four along upper lip, fifth posterior to rictus, three pores behind eye. Frontal pore absent. Preopercular canal with three pores. Mandibular canal with seven pores, six along lower jaw and the last well posterior to rictus. Supratemporal commissure with three pores, the median pore minute. Predorsal vertebrae 7–10; preanal vertebrae 26–36; precaudal vertebrae 33–42; total vertebrae 120–127.

***Teeth*** small, conical to blunt (Fig. 8). Intermaxillary teeth arranged in ~ 3–5 transverse rows, continuous with maxillary and vomerine tooth patches. Maxillary and mandibular teeth in bands, broader anteriorly with ~ 2–3 rows, narrowing posteriorly to ~ 1–2 rows. Vomerine teeth blunt, forming an elongate patch of approximately three transverse rows; in larger specimens (>45 cm TL), vomerine tooth patch broader, comprising ~ 4–5 rows (Fig. 8: B3).

##### Coloration.

When fresh (Fig. 2A, B), region above the lower margin of the eye, including the dorsal surface of the head, and the body above the pectoral fin dark brown. Fleshy tip of snout pale yellowish green. Upper and lower lips light brown. Pectoral fin and lateral line pores pale, with an indistinct silvery band below the lateral line pores. Region below the lower margin of the eye, including the ventral surface of the head, and the ventral surface of the body below the pectoral fin pale white. Vertical fins pale with black margins. Peritoneum pale.

##### Size.

Maximum observed total length 483 mm. Individuals exceeding 300 mm TL were primarily collected from northeastern Taiwan, whereas specimens smaller than 300 mm TL were obtained from both northeastern and southwestern waters of Taiwan.

##### Distribution.

Known from southwestern Japan and extending southward to the South China Sea near Luzon Island, Philippines, at depths of ~ 100–200 m. In Taiwanese waters, the species has been recorded from northeastern, western, and southwestern regions.

##### Remarks.

The validity of *Gnathophis heterognathos* and the treatment of *G. nystromi* as its junior synonym were proposed by Castle (1963) and Karmovskaya (2004). Specimens identified as *G. nystromi* from Japanese waters closely resemble Taiwanese specimens of *G. heterognathos* in most characters. As indicated by the data presented in Tables 2, 4, specimens from Taiwan are also similar in most characters to the holotype of *Myrophis heterognathos* (BMNH 1867.11.28.305). Based on these observations, the present study concurs with previous authors in recognizing *G. nystromi* as a junior synonym of *G. heterognathos*. Larger specimens were mainly collected from northeastern Taiwan, and two specimens (TOU-AE 9699, TOU-AE 9702) are ripe females with well-developed ovaries containing loose eggs, suggesting they were approaching spawning. Among the examined species of *Gnathophis*, ontogenetic variation in dentition was observed only in *G. heterognathos*. Specimens exceeding ~ 45 cm TL showed distinct changes in the morphology and/or arrangement of the vomerine teeth. In contrast, no ontogenetic changes in dentition were detected in the other four species examined.

#### Gnathophis kbalanensis

Taxon classificationAnimaliaAnguilliformesCongridae

Huang, Liao, Chen & Chan sp. nov.

961DEDE7-2236-5049-93E9-E4772C31A2D1

https://zoobank.org/4A26E6FD-0C14-4E0A-9B10-F7540D2CB18C

**Figure 3.**
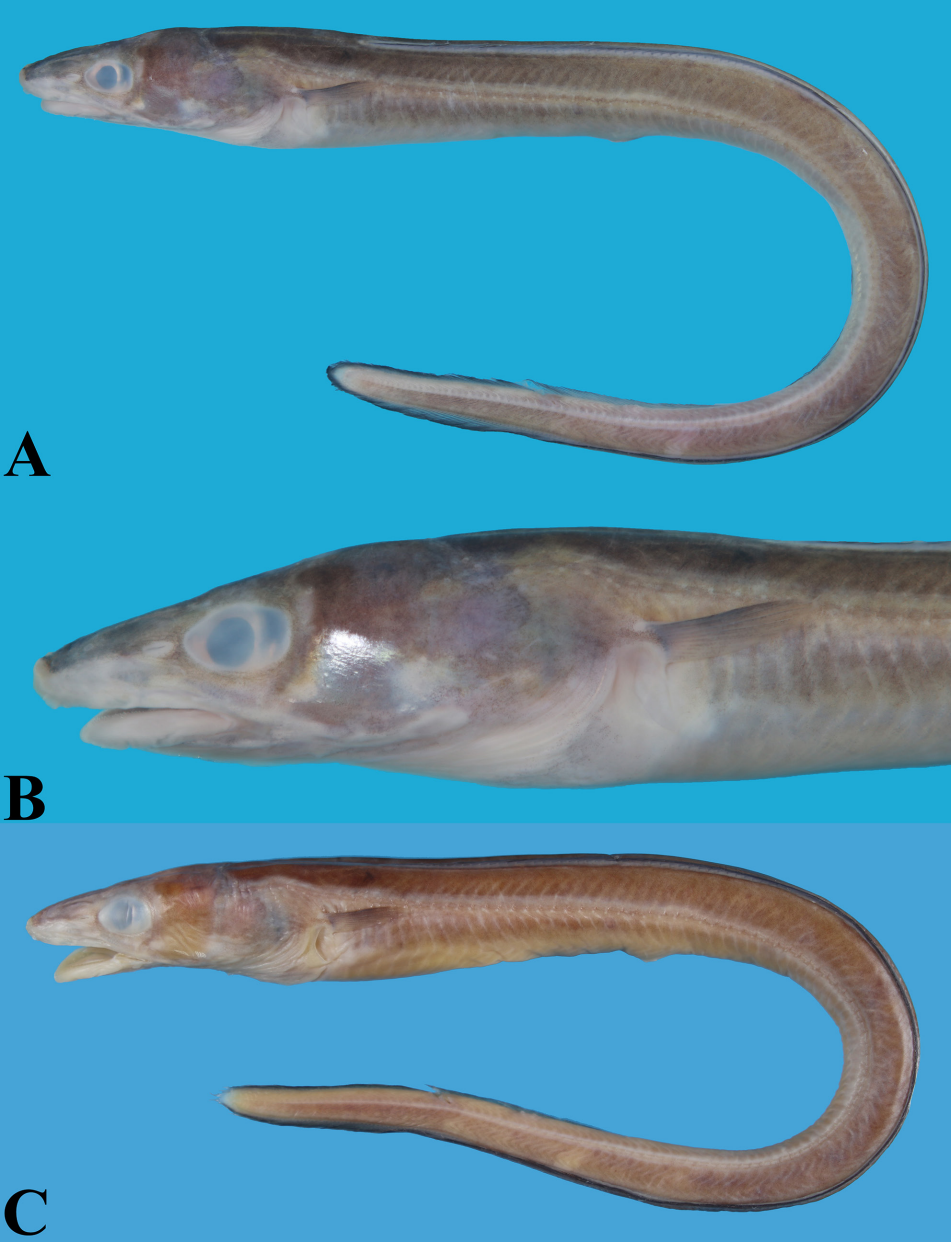
*Gnathophis kbalanensis* sp. nov. Holotype, TOU-AE 9480, 472 mm TL. **A**. Lateral view; **B**. Lateral view of anterior portion of head; **C**. Preserved.

Figs 3, 6, 7; Tables 1, 2, 4, 6, 8–12

##### English name.

Kbalan’s conger.

##### Chinese name.

噶瑪蘭頜吻糯鰻.

##### Type material.

***Holotype***. • TOU-AE 9480, 472 mm TL, a ripe female with free eggs, off Daxi, Yilan, northeastern Taiwan, 23 Mar. 2023, bottom trawl, ca 300 m, coll. J.-F. Huang. ***Paratypes***. • 3 specimens, 363–443 mm TL. TOU-AE 9130, 375 mm TL, a ripe female and free eggs, off Daxi, Yilan, northeastern Taiwan, 22 Dec. 2022, bottom trawl, ca 300 m, coll. J.-F. Huang. • TOU-AE 9594, 443 mm TL, a ripe female and free eggs, off Daxi, Yilan, northeastern Taiwan, 18 Feb. 2023, bottom trawl, ca 300 m, coll. J.-F. Huang. • TOU-AE 10231, 363 mm TL, off Daxi, Yilan, northeastern Taiwan, 18 Jan. 2024, bottom trawl, ca 300 m, coll. J.-F. Huang.

##### Diagnosis.

This species can be distinguished from all other congeners possessing elevated lateral line pores above the pectoral fin by the following combination of characters: a pale peritoneum; head length 16.4–17.6% TL; trunk length 21.0–23.1% TL; precaudal vertebrae 42–43, total vertebrae 134–137; preanal lateral line pores 32–35; and POP pores three.

##### Description.

Measurements and counts in Tables 4, 6. ***Body*** moderately elongate, cylindrical in cross section anteriorly, becoming progressively compressed posteriorly. Tail more than half of total length, 0.6× TL, tip slightly rounded. Preanal length ~ 0.4× TL; trunk length 0.3–0.4× tail length. Dorsal fin origin slightly anterior to tip of pectoral fin; in some smaller paratypes, dorsal fin origin located above middle pectoral fin; dorsal, caudal, and anal fins continuous around tip of tail. Predorsal length ~ 0.2× TL. Anal fin originating immediately posterior to anus. Pectoral fin well developed, distally pointed, with narrow base. Gill opening relatively small, smaller than eye diameter; upper end nearly opposite middle of pectoral fin base; interbranchial width wider than gill opening and eye diameter.

**Table 4.** Morphometric data of *Gnathophis kbalanensis* sp. nov. and *G. melanurum* sp. nov. from Taiwan.

	***G. kbalanensis* sp. nov**.		***G. melanurum* sp. nov**.	
	**Holotype**	**Paratypes**	**Holotype**	**PT+NT**
Total length (mm)	472	363–443 (*n* = 3)	300	363–443 (*n* = 49)
As % of TL		Mean (Range)		Mean (Range)
Head length	17.6	17.0 (16.4–17.6)	16.4	17.2 (16.1–18.7)
Predorsal length	21.0	19.1 (18.2–21.0)	17.9	18.8 (17.6–20.0)
Preanal length	38.6	39.0 (38.3–39.7)	38.3	38.0 (34.8–40.0)
Trunk length	21.0	22.0 (21.0–23.1)	22.0	20.7 (17.8–23.0)
Tail length	61.4	61.0 (60.3–61.7)	61.7	62.0 (60.0–65.2)
Depth at gill opening	6.8	6.7 (6.3–7.0)	6.1	6.2 (5.0–7.4)
Depth at anus	5.9	5.6 (5.2–5.9)	6.3	5.6 (3.8–7.2)
As % of PAL				
Head length	45.6	43.6 (41.6–45.6)	42.7	45.4 (42.6–49.1)
Trunk length	54.4	56.4 (54.4–58.4)	57.3	54.6 (50.9–57.4)
Predorsal length	54.5	48.9 (46.7–54.5)	46.6	49.5 (46.1–53.2)
Depth at gill opening	17.7	17.0 (15.9–17.7)	15.9	16.3 (12.7–18.5)
Depth at anus	15.4	14.4 (13.6–15.4)	16.5	14.7 (10.6–18.4)
As % of HL				
Snout length	29.3	30.2 (29.3–30.9)	29.8	29.4 (26.8–32.3)
Eye diameter	17.8	17.5 (17.2–17.8)	22.4	21.5 (18.1–25.0)
Interorbital width	15.7	13.9 (11.7–15.7)	6.1	7.6 (5.6–9.6)
Interbranchial width	25.6	27.5 (25.3–29.6)	28.4	25.3 (16.9–31.0)
Gill opening width	13.9	13.0 (10.7–14.1)	12.8	11.4 (8.7–16.2)
Upper jaw length	32.8	34.0 (32.4–36.6)	32.9	35.6 (32.7–38.0)
Lower jaw length	27.3	27.9 (26.3–29.7)	25.7	28.7 (25.3–31.0)
Pectoral-fin length	29.3	29.1 (26.9–30.3)	34.1	32.5 (29.2–36.9)

***Head*** moderately large, reflecting relatively stout body, deepest at approximately gill opening. Head length 0.7–0.8× trunk length and 0.4–0.5× preanal length. Snout long and pointed, 1.6–1.8× eye diameter, projecting distinctly beyond lower jaw; lower jaw clearly shorter than snout. Fleshy portion of snout projecting anteriorly beyond anterior margin of intermaxillary tooth patch. Rictus below anterior half of eye. Anterior nostril tubular, near tip of snout, directed ventrolaterally. Posterior nostril elliptical, located anterior to eye at mid-eye level. Upper lip with shallow, free, upturned flange beginning at second infraorbital pore and ending below middle of eye; lower lip with well-developed downturned flange.

***Lateral line*** complete, first pore on each side slightly enlarged, canal extending to caudal fin base; 6–7 pores before pectoral-fin base, 7–11 before dorsal fin origin, and 32–35 before anal fin origin; pores above pectoral fin elevated from pores 7/8–13/14, second pore also slightly elevated.

***Head pores*** small (Fig. 6C). Supraorbital canal with six pores: first (ethmoidal) and second on ventral surface of snout tip just anterior to lip; third on dorsal surface of snout tip; fourth on dorsal surface of anterior half of snout; fifth above anterior margin of eye; sixth above posterior margin of eye. Infraorbital canal with eight pores, first four along upper lip, fifth posterior to rictus, and three posterior to eye. No frontal pore. Preopercular canal with three pores. Mandibular canal with seven pores, six along lower jaw and last well posterior to rictus. Supratemporal commissure with three pores, median pore minute. Predorsal vertebrae 9–12; preanal vertebrae 34–36; precaudal vertebrae 42–43; total vertebrae 134–137.

***Teeth*** small, conical to blunt (Fig. 7B). Intermaxillary teeth in ~ 4–5 transverse rows, continuous with maxillary and vomerine teeth. Maxillary and mandibular teeth arranged in bands, broader anteriorly (~ 4–5 rows) and narrower posteriorly (~ 2–3 rows). Vomerine teeth blunt, forming an elongate patch with multiple, irregular rows.

##### Coloration.

When fresh (Fig. 3A, B), the region from the mouth cleft to the dorsal surface of the head, the upper three-quarters of the body before the anus, and the upper half of the body posterior to the anus are brown. The fleshy tip of the snout is pale yellowish green. The posterior nostril, lateral line pores, upper and lower lips, area surrounding the gill opening, and ventral surface of the head are white. The ventral surface of the preanal region and the lower half of the postanal body are grayish white. Color of peritoneum, vertical fins are black. Black pigmentation present on the pectoral fin. After preservation (Fig. 3C), areas surrounding the upper and lower lips pale whitish. Dorsal surface of head and the region from the posterior margin of the eye to the pectoral fin base light brown. Areas surrounding the lateral line pores pale. Upper three-quarters of the preanal body dark brown, the postanal body gradually fading to light brown posteriorly. Abdomen light brown. Dorsal, anal, and caudal fins black.

##### Size.

Largest specimen examined 472 mm TL.

##### Etymology.

The specific name *kbalanensis* is derived from the type locality “Kbalan”, an ancient name of the Yilan region (Kat-má-lán in Taiwanese or Cabaran in Spanish), used ~ 800–1300 years ago.

##### Distribution.

Known only from the type series collected from northeastern Taiwan at depths of ~ 200–300 m.

##### Remarks.

*Gnathophis kbalanensis* closely resembles *G. heterognathos* in most morphometric characters but can be distinguished by vertebral counts. *Gnathophis kbalanensis* has more precaudal vertebrae (42–43 vs 33–42) and more total vertebrae (134–137 vs 120–127). In addition, below the lateral-line pores, *G. heterognathos* has a bright silvery stripe, whereas *G. kbalanensis* lacks this feature. The pectoral fin of *G. heterognathos* is pale, whereas *G. kbalanensis* exhibits darker pigmentation on the pectoral fin. All specimens of *G. kbalanensis* were collected from northeastern Taiwan. Three specimens (TOU-AE 9480, TOU-AE 9130, TOU-AE 9594) are ripe females with well-developed ovaries and loosely attached eggs, indicating imminent spawning.

#### Gnathophis melanurum

Taxon classificationAnimaliaAnguilliformesCongridae

Huang, Liao, Chen & Chan sp. nov.

49A56CED-F38B-5C48-A808-2A5B26E79CB7

https://zoobank.org/50F098C1-9BD7-435E-899B-6639D2431866

**Figure 4.**
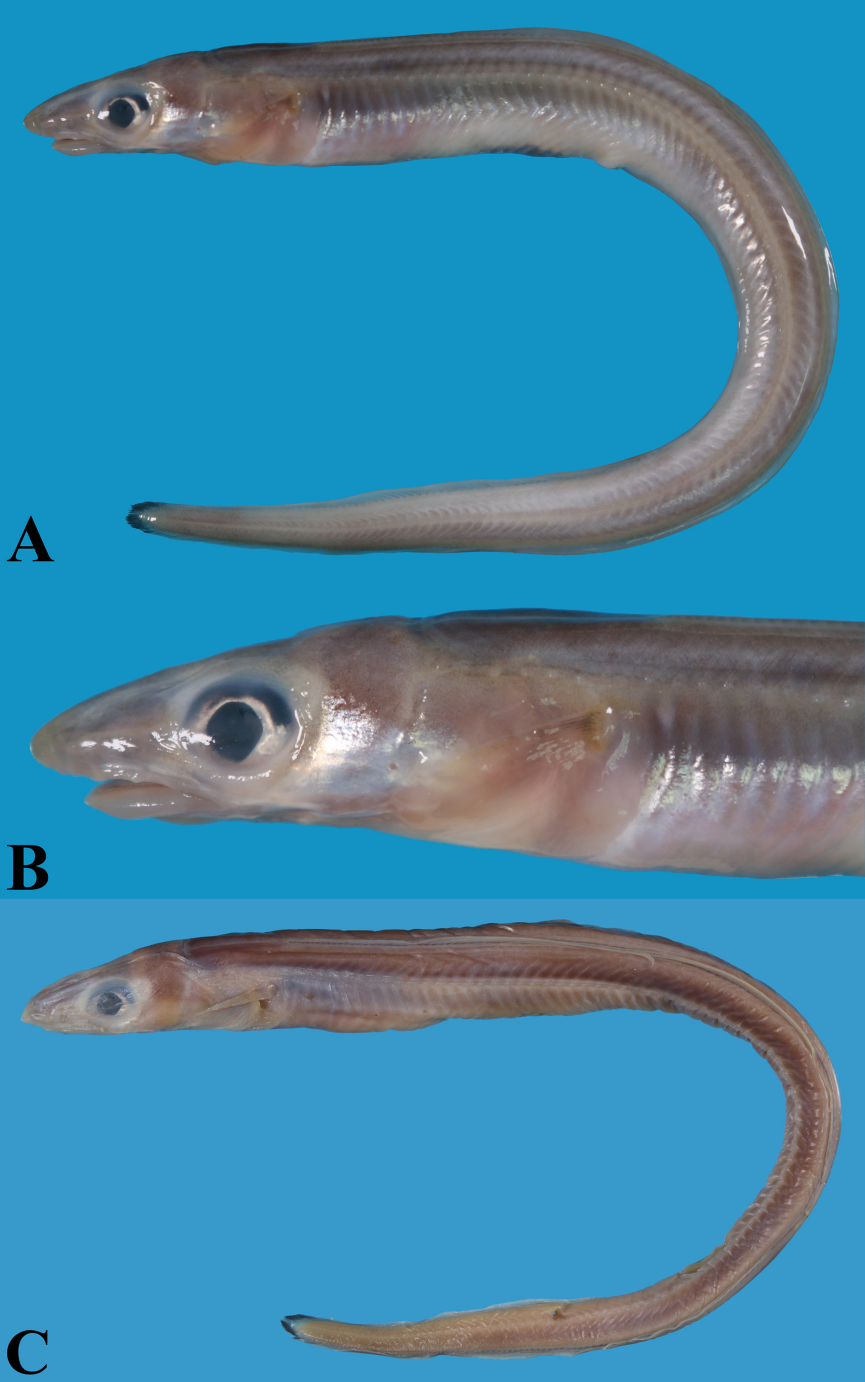
*Gnathophis melanurum* sp. nov. Holotype, TOU-AE 8777, 300 mm TL. **A**. Lateral view; **B**. Lateral view of anterior portion of head; **C**. Preserved.

Figs 4, 6, 7; Tables 1, 2, 4, 6, 8–12

##### English name.

Black tail conger.

##### Chinese name.

黑尾頜吻糯鰻.

##### Type material.

***Holotype***. • TOU-AE 8777, 300 mm TL, a ripe female with free eggs, off Daxi, Yilan, northeastern Taiwan, 27 Aug. 2022, bottom trawl, ca 300 m, coll. J.-F. Huang. ***Paratypes***. • 46 specimens, 162–353 mm TL. • **Daxi, Yilan, northeastern Taiwan**. TOU-AE 8778, 325 mm TL, TOU-AE 8779, 281 mm TL, 27 Aug. 2022, bottom trawl, ca 300 m, coll. J.-F. Huang. • TOU-AE 8875, 273 mm TL, TOU-AE 8876, 275 mm TL, TOU-AE 8877, 322 mm TL, a ripe female and free eggs, TOU-AE 8878, 332 mm TL, TOU-AE 8879, 342 mm TL, a ripe female and free eggs, a ripe female and free eggs, 01 Oct. 2022, bottom trawl, ca 300 m, coll. J.-F. Huang. • TOU-AE 9325, 277 mm TL, 14 Jan. 2023, bottom trawl, ca 300 m, coll. J.-F. Huang. • TOU-AE 9129, 295 mm TL, 22 Dec. 2022, bottom trawl, ca 300 m, coll. J.-F. Huang. • TOU-AE 9507, 341 mm TL, TOU-AE 9508, 353 mm TL, 28 Feb. 2023, bottom trawl, ca 300 m, coll. J.-F. Huang. TOU-AE 9465, 313 mm TL, a ripe female and free eggs, 15 Mar. 2023, bottom trawl, ca 300 m, coll. J.-F. Huang. TOU-AE 9427, 260 mm TL, 20 Aug. 2022, bottom trawl, ca 300 m, coll. J.-F. Huang. • TOU-AE 9360, 352 mm TL, TOU-AE 9361, 298 mm TL, TOU-AE 9362, 296 mm TL, TOU-AE 9363, 285 mm TL, TOU-AE 9364, 282 mm TL, TOU-AE 9365, 276 mm TL, TOU-AE 9366, 280 mm TL, TOU-AE 9367, 272 mm TL, TOU-AE 9368, 258 mm TL, TOU-AE 9369, 258 mm TL, 30 Jan. 2023, bottom trawl, ca 300 m, coll. J.-F. Huang. • TOU-AE 10438, 212 mm TL, 18 May. 2024, bottom trawl, ca 300 m, coll. J.-F. Huang. TOU-AE 10217, 162 mm TL, 17 Jan. 2024, bottom trawl, ca 300 m, coll. J.-F. Huang. • TOU-AE 10762, 315 mm TL, a ripe female and free eggs, TOU-AE 10763, 321 mm TL, a ripe female and free eggs, TOU-AE 10764, 339 mm TL, a ripe female and free eggs, 01 Dec. 2024, bottom trawl, ca 300 m, coll. J.-F. Huang. • TOU-AE 9885, 313 mm TL, TOU-AE 9886, 308 mm TL, 03 Jun. 2023, bottom trawl, ca 300 m, coll. J.-F. Huang. • TOU-AE 9901, 245 mm TL, TOU-AE 9902, 251 mm TL, TOU-AE 9904, 240 mm TL, TOU-AE 9905, 248 mm TL, TOU-AE 9906, 260 mm TL, TOU-AE 9907, 283 mm TL, TOU-AE 9908, 294 mm TL, 08 Jun. 2023, bottom trawl, ca 300 m, coll. J.-F. Huang. • TOU-AE 10866, 265 mm TL, TOU-AE 10867, 268 mm TL, 07 Jan. 2025, bottom trawl, ca 300 m, coll. J.-F. Huang. • NMMB-P34411, 311 mm TL, NMMB-P34419, 2 specimens, 315–321 mm TL, 21 Jun. 2020, bottom trawl, ca 300 m, coll. N.-W. Lai. • **Badouzhi, Keelung, northeastern Taiwan**. TOU-AE 9806, 335 mm TL, 15 May. 2023, bottom trawl, ca 300 m, coll. J.-F. Huang. • **Dong-gang, Pingtung, southwestern Taiwan**. NMMB-P28484, 178 mm TL, 01 Jan. 2018, bottom trawl, ca 300 m, coll. H.-C. Ho. • TOU-AE 8006, 193 mm TL, TOU-AE 8007, 200 mm TL, 24 Jan. 2021, bottom trawl, ca 300 m, coll. J.-F. Huang.

##### Additional material.

• 3 specimens, 243+–288+ mm TL. TOU-AE 9887, 288+ mm TL, Daxi, Yilan, 03 Jun. 2023, bottom trawl, ca 300 m, coll. J.-F. Huang. • TOU-AE 9903, 243+ mm TL, Daxi, Yilan, 08 Jun. 2023, bottom trawl, ca 300 m, coll. J.-F. Huang. • NMMB-P11155, 284+ mm TL, Daxi, Yilan, 08 Dec. 2010, bottom trawl, ca 300 m, coll. H.-C. Ho.

##### Diagnosis.

This species can be distinguished from all other congeners possessing elevated lateral line pores above the pectoral fin by the following combination of characters: dorsal and anal fins pale, whereas caudal fin black; peritoneum black; preanal vertebrae 28–32; precaudal vertebrae 37–41; total vertebrae 120–125; preanal lateral line pores 29–32; and POP pores three.

##### Description.

Measurements and counts in Tables 4, 6. ***Body*** moderately elongate, cylindrical in cross section anteriorly, becoming laterally compressed posteriorly. Tail more than half of total length, 0.6–0.7× total length (TL), tip slightly rounded. Preanal length 0.3–0.4 TL. Trunk length 0.3–0.4× tail length. Dorsal fin origin slightly posterior to the base of the pectoral fin; in smaller paratypes, dorsal fin origin located between slightly posterior to the pectoral fin base and above the middle of the pectoral fin; dorsal, caudal, and anal fins continuous around the tip of the tail. Predorsal length ~ 0.2 TL. Anal fin originating immediately posterior to the anus. Pectoral fin well developed, distally pointed, with a narrow base. Gill opening relatively small, smaller than eye diameter, its upper margin nearly opposite the middle of the pectoral fin base; interbranchial width broader than gill opening and eye.

***Head*** moderately large, reflecting less slender body, deepest at approximately the level of the gill opening. Head length 0.7–1.0× trunk length and 0.4–0.5× preanal length. Snout long and pointed, 1.2–1.7× eye diameter, projecting well beyond lower jaw; lower jaw distinctly shorter than snout. Fleshy portion of snout projecting anteriorly beyond the anterior margin of the intermaxillary tooth patch. Rictus located below anterior half of the eye. Anterior nostril tubular, near tip of snout, directed ventrolaterally. Posterior nostril elliptical, situated anterior to eye at mid-eye level. Upper lip with a shallow, free, upturned flange, beginning at second infraorbital pore and ending below the middle of the eye. Lower lip with a well-developed, downturned flange.

***Lateral line*** complete; first pore on each side slightly enlarged; canal extending to base of caudal fin. 5–7 pores before pectoral-fin base, 6–10 before dorsal fin origin, and 29–32 before anal fin origin. Lateral line pores above pectoral fin elevated, extending approximately from pores 6–8 anteriorly to pores 12–18 posteriorly; second pore also slightly elevated.

***Head pores*** small (Fig. 6D). Supraorbital canal with six pores: first (ethmoidal pore) and second on ventral surface of snout tip just anterior to lip; third on dorsal surface of snout tip; fourth on dorsal surface of anterior half of snout; fifth above anterior margin of eye; sixth above posterior margin of eye. Infraorbital canal with eight pores, first four along upper lip, fifth posterior to rictus, and three pores posterior to eye. Frontal pore absent. Preopercular canal with three pores. Mandibular canal with seven pores, six along lower jaw and last well posterior to rictus. Supratemporal commissure with three pores, the median pore minute. Predorsal vertebrae 8–11; preanal vertebrae 28–32; precaudal vertebrae 37–41; total vertebrae 120–125.

***Teeth*** small, conical to blunt (Fig. 7A). Intermaxillary teeth arranged in approximately four transverse rows, continuous with maxillary and vomerine tooth patches. Maxillary and mandibular teeth in bands, broader anteriorly with ~ 2–3 rows, narrowing posteriorly to ~ 1–2 rows. Vomerine teeth blunt, forming an elongate patch of ~ 3–4 transverse rows.

##### Coloration.

When fresh (Fig. 4A, B), dorsal surface of head and upper half of body light brown, sometimes yellowish green; fleshy tip of snout pale yellowish green. Ventral surface of head, pectoral fin, and area surrounding gill opening pale. Lateral line pores white, with an indistinct silvery band below the lateral line pores. Ventral surface of body pale white. Dorsal and anal fins pale, whereas caudal fin black. Peritoneum black. After preservation (Fig. 4C), Areas surrounding the upper and lower lips pale whitish. Dorsal surface of head light brown. Lateral line pores and surrounding areas pale. Body dark brown above the lateral line pores and light brown below them, including the ventral surface. Dorsal and anal fins pale, whereas caudal fin black.

##### Size.

Largest specimen examined 353 mm TL.

##### Etymology.

The specific name *melanurum* is derived from the Greek *melan* (black) and *urum* (tail), in reference to the black caudal fin.

##### Distribution.

Known from the type series collected from northeastern and southwestern waters off Taiwan, at depths of ~ 200–300 m.

##### Remarks.

*Gnathophis melanurum* resembles *G. heterognathos* in most morphometric characters but differs clearly in coloration of the vertical fins. In *G. melanurum*, the dorsal and anal fins are pale and the caudal fin is black, whereas in *G. heterognathos* all vertical fins are pale with black margins. Larger specimens were mainly collected from northeastern Taiwan. Seven specimens (TOU-AE 8777, TOU-AE 8877, TOU-AE 8878, TOU-AE 8879, TOU-AE 9465, TOU-AE 10762, TOU-AE 10763, TOU-AE 10764) are ripe females with well-developed ovaries containing loosely attached eggs, indicating imminent spawning.

#### Gnathophis nanhaiensis

Taxon classificationAnimaliaAnguilliformesCongridae

Huang, Chen & Chan sp. nov.

C706300B-5C74-5F39-AA0B-46743604991B

https://zoobank.org/ADE1B3A5-7EBA-4586-A73E-EB2718CADC2D

**Figure 5.**
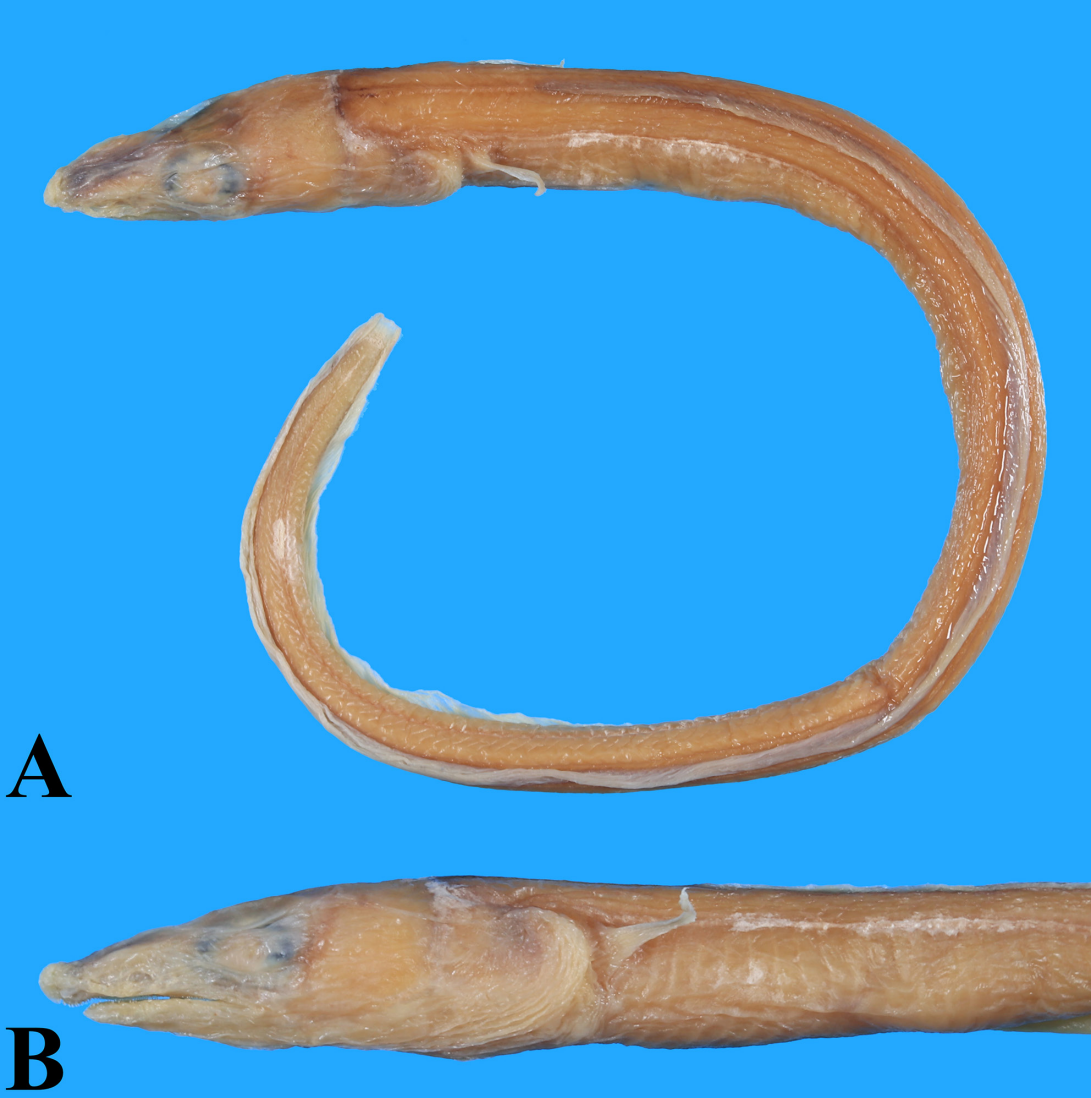
*Gnathophis nanhaiensis* sp. nov. Holotype, ASIZP66800, 245 mm TL. **A**. Lateral view; **B**. Lateral view of anterior portion of head.

**Figure 6.**
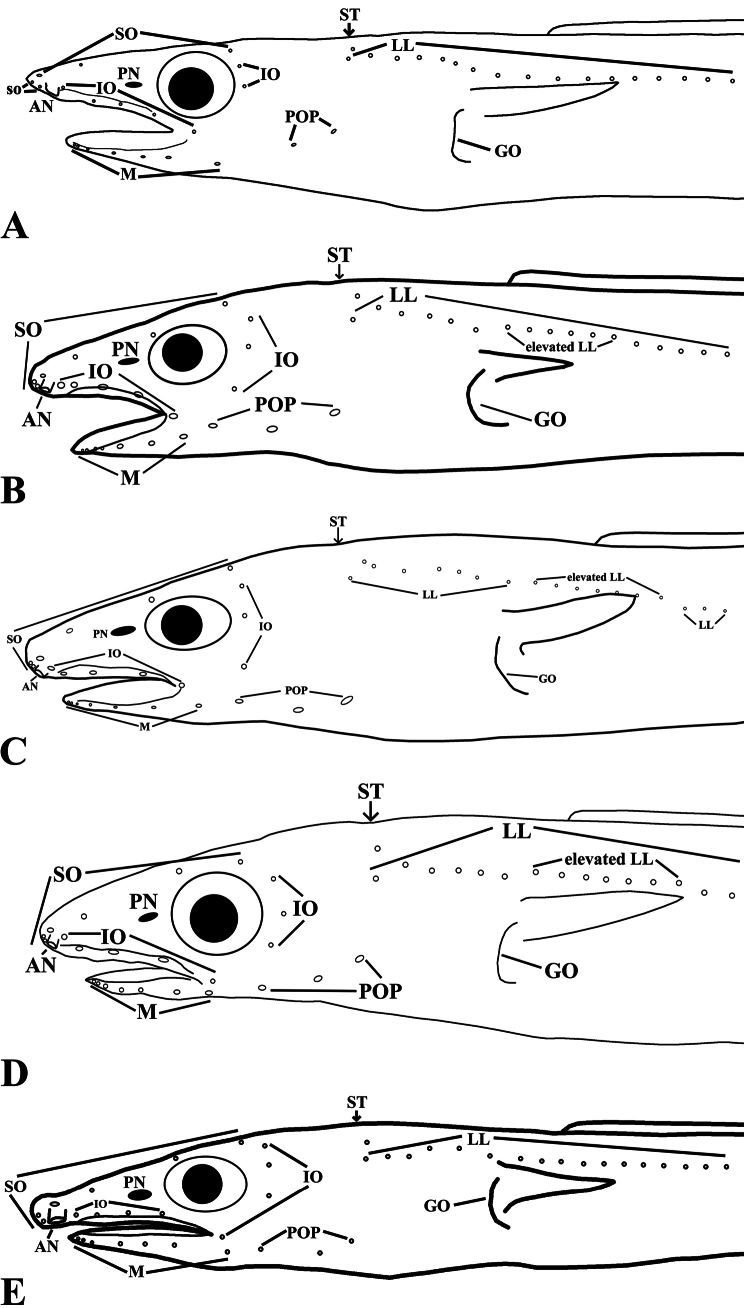
Lateral view of head showing the head pores and anterior lateral-line pores, arrow indicates the ST pore. **A**. *Gnathophis asanoi* Karmovskaya, 2004, NMMB-P30378, 350 mm TL; **B**. *Gnathophis heterognathos* (Bleeker, 1858), TOU-AE 9900, 329 mm TL; **C**. *Gnathophis kbalanensis* sp. nov. Holotype, TOU-AE 9480, 472 mm TL; **D**. *Gnathophis melanurum* sp. nov. Holotype, TOU-AE 8777, 300 mm TL; **E**. *Gnathophis nanhaiensis* sp. nov. Holotype, ASIZP66800, 245 mm TL.

**Figure 7.**
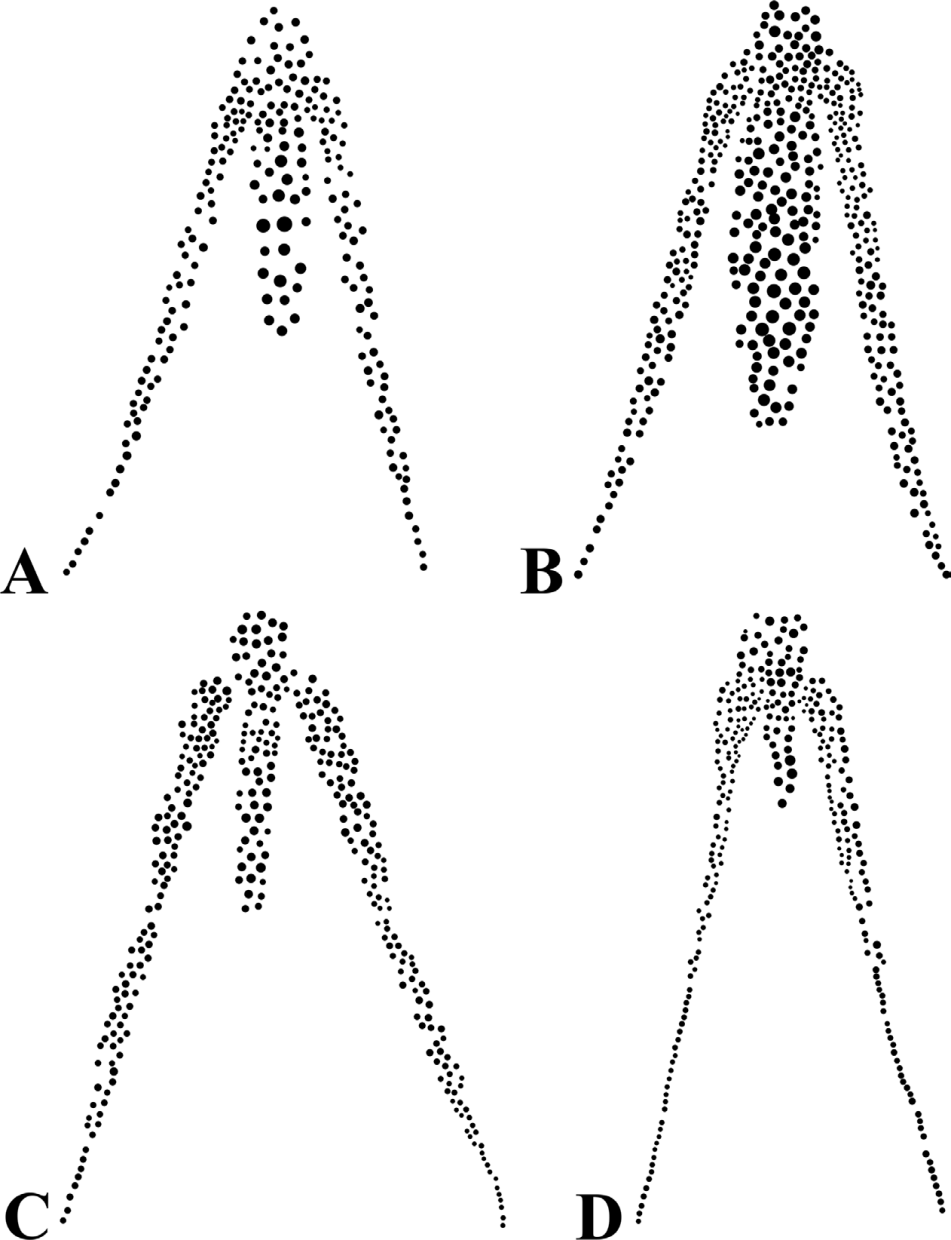
Tooth pattern on upper jaw. **A**. *Gnathophis melanurum* sp. nov., TOU-AE 8777, 300 mm TL, holotype; **B**. *Gnathophis kbalanensis* sp. nov., TOU-AE 9480, 472 mm TL, holotype; **C**. *Gnathophis asanoi*, TOU-AE 6842, 357 mm TL; **D**. *Gnathophis nanhaiensis* sp. nov., ASIZP66800, 245 mm TL, holotype. Not to scale.

Figs 5, 6, 7; Tables 5, 7–11, 13

##### English name.

Nan Hai’s conger.

##### Chinese name.

南海頜吻糯鰻.

##### Type material.

***Holotype***. • ASIZP66800, 245 mm TL, CP315, 21°67'01"N, 117°71'95"E, South China Sea, 17 Aug. 2005, bottom trawl, 509 m, coll. S.-M. Yeh. ***Paratype***. • FRIP00480, 275 mm TL, Taiwan, 540 m depth, no data available on fishing method, coll. D.-A. Lee.

##### Diagnosis.

This species differs from all congeners of *Gnathophis* lacking elevated lateral line pores above the pectoral fin by the following combination of characters: vertical fins and peritoneum pale; head length 16.9–17.9% TL; preanal length 42.0–43.6% TL; predorsal length 19.7–20.3% TL; trunk length 24.1–26.7% TL; precaudal vertebrae 47–48; total vertebrae 139–143; and POP pores three.

##### Description.

Measurements and counts in Tables 5, 7. ***Body*** moderately elongate, cylindrical in cross section anteriorly, becoming laterally compressed posteriorly. Tail more than half of total length, ~ 0.6× TL, tip slightly rounded. Preanal length ~ 0.4 TL. Trunk length 0.4–0.5× tail length. Dorsal fin origin anterior to tip of pectoral fin, continuous around tip of tail with the caudal and anal fins. Predorsal length ~ 0.2 TL. Anal fin originating immediately posterior to the anus. Pectoral fin well developed, distally pointed, with a narrow base. Gill opening relatively small, smaller than eye diameter, its upper margin nearly opposite the middle of the pectoral fin base; interbranchial width broader than gill opening and eye.

**Table 5.** Morphometric data of *Gnathophis nanhaiensis* sp. nov. and *G. asanoi* from Taiwan.

	***Gnathophis nanhaiensis* sp. nov**.		***Gnathophis asanoi***
	**Holotype**	**Paratype**	
Total length (mm)	245	275	290–357 (*n* = 3)
%TL			Mean (Range)
Head length	17.9	16.9	14.3 (13.9–15.0)
Predorsal length	20.3	19.7	16.5 (16.0–16.9)
Preanal length	42.0	43.6	36.4 (35.2–38.1)
Trunk length	24.1	26.7	22.1 (20.2–24.0)
Tail length	58.0	56.4	63.6 (61.9–64.8)
Depth at gill opening	4.8	5.7	4.6 (4.2–5.0)
Depth at anus	4.3	4.7	4.1 (3.6–4.4)
As % of PAL			
Head length	42.6	38.8	39.4 (37.0–42.6)
Trunk length	57.4	61.2	60.6 (57.4–63.0)
Predorsal length	48.4	45.1	45.3 (44.4–47.0)
Depth at gill opening	11.5	13.2	12.6 (12.0–13.9)
Depth at anus	10.2	10.8	11.2 (10.3–12.3)
%HL			
Snout length	28.8	30.1	30.0 (27.0–31.4)
Eye diameter	19.4	17.8	17.8 (16.9–19.0)
Interorbital width	6.5	4.6	7.2 (5.0–8.7)
Interbranchial width	19.2	26.6	22.9 (19.7–24.7)
Gill opening width	9.5	13.2	12.7 (11.9–14.0)
Upper jaw length	38.3	38.3	37.2 (36.8–37.4)
Lower jaw length	30.5	30.2	28.0 (26.1–31.3)
Pectoral-fin length	24.5	30.4	26.0 (24.9–26.6)

**Table 6.** Meristic data of *Gnathophis heterognathos*, *Myrophis heterognathos*, *G. kbalanensis* sp. nov., and *G. melanurum* sp. nov.

	***G. heterognathos***	***Myrophis heterognathos***	***G. kbalanensis* sp. nov**.		***G. melanurum* sp. nov**.	
		**BMNH 1867.11.28.305**	**Holotype**	**Paratypes**	**Holotype**	**Paratypes + nontypes**
Total length (mm)	155+–483 (*n* = 70)	137.3	472	363–443 (*n* = 3)	300	162–353 (*n* = 49)
Meristics						
Predorsal vertebrae	7–10	10	12	9–10	9	8–11
Preanal vertebrae	26–36	29	34	34–36	32	28–32
Precaudal vertebrae	33–42	39	43	42–43	40	37–41
Total vertebrae	78+–127	122	136	134–137	125	98+–125
Prepectoral-fin pores	5–8	-	7	6	6	5–7
Predorsal pores	7–11	-	11	7–8	8	6–10
Preanal pores	22–35	31	32	32–35	31	29–32
SO	6	6	6	6	6	6
IO	3+1+4	8	3+1+4	3+1+4	3+1+4	3+1+4
POM	7+3	10	7+3	7+3	7+3	7+3
ST	3	3	3	3	3	3

**Table 7.** Meristic data of *Gnathophis nanhaiensis* sp. nov. and *G. asanoi* from Taiwan.

	***Gnathophis nanhaiensis* sp. nov**.		***Gnathophis asanoi***
	**Holotype**	**Paratype**	
Total length (mm)	245	275	290–357 (*n* = 3)
Meristics			
Predorsal vertebrae	11	10	9–11
Preanal vertebrae	40	42	34–37
Precaudal vertebrae	47	48	42–44
Total vertebrae	139	143	139–141
Prepectoral-fin pores	6	7	6–7
Predorsal pores	9	11	9–10
Preanal pores	37	41	35–37
SO	6	6	5
IO	3+1+4	3+1+4	3+1+4
POM	7+3	7+3	7+2
ST	3	3	3

***Head*** moderately large, reflecting less slender body, deepest at approximately level of gill opening. Head length 0.6–0.7× trunk length and ~ 0.4× preanal length. Snout long and pointed, 1.5–1.7× eye diameter, projecting well beyond lower jaw; lower jaw distinctly shorter than snout. Fleshy portion of snout projecting anteriorly beyond the anterior margin of the intermaxillary tooth patch. Rictus located below anterior half of eye. Anterior nostril tubular, near tip of snout, directed ventrolaterally. Posterior nostril elliptical, situated anterior to eye at mid-eye level. Upper lip with a shallow, free, upturned flange, beginning at second infraorbital pore and ending below the middle of eye. Lower lip with a well-developed, downturned flange.

***Lateral line*** complete; first pore on each side slightly enlarged; canal extending to base of caudal fin. 6–7 before pectoral-fin base, 9–11 before the dorsal fin origin, and 37–41 before anal fin origin; lateral line pores above pectoral fin not elevated.

***Head pores*** small (Fig. 6E). Supraorbital canal with six pores: first (ethmoidal pore) and second on ventral surface of snout tip just anterior to the lip; third on dorsal surface of snout tip; fourth on dorsal surface of anterior half of snout; fifth above anterior margin of eye; sixth above posterior margin of eye. Infraorbital canal with eight pores, first four along the upper lip, fifth posterior to rictus, and three pores posterior to eye. Frontal pore absent. Preopercular canal with three pores. Mandibular canal with seven pores, six along lower jaw and the last well posterior to rictus. Supratemporal commissure with three pores, the median pore minute. Predorsal vertebrae 10–11; preanal vertebrae 40–42; precaudal vertebrae 47–48; total vertebrae 139–143.

***Teeth*** small, conical to blunt (Fig. 7D). Intermaxillary teeth arranged in approximately four transverse rows, continuous with maxillary and vomerine tooth patches. Maxillary and mandibular teeth in bands, broader anteriorly with approximately 4–5 rows, narrowing posteriorly to a single row. Vomerine teeth blunt, forming a moderately elongate patch of ~ 2–3 transverse rows.

**Figure 8.**
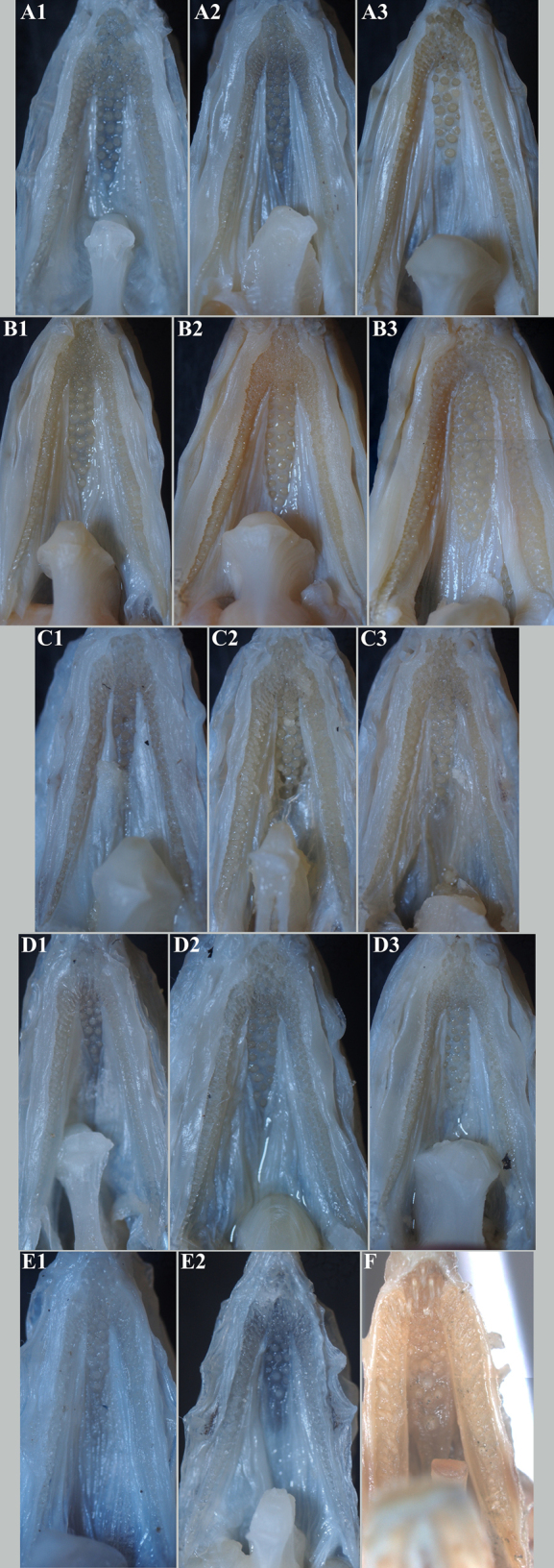
Vomerine tooth pattern and variation of *Gnathophis heterognathos*. From the left to right: **A1–A3**. Collected from Daxi, Yilan, TOU-AE 10869, 173 mm TL, TOU-AE 8031, 251 mm TL, TOU-AE 9900, 329 mm TL; **B1–B3**. Collected from Badouzhi, Keelung, TOU-AE 9845, 246 mm TL, TOU-AE 9837, 355 mm TL, TOU-AE 9832, 483 mm TL; **C1–C3**. Collected from Xianxi, Changhua, TOU-AE 8021, 180 mm TL, TOU-AE 8180, 234 mm TL, TOU-AE 8181, 295 mm TL; **D1–D3**. Collected from Ke-tzu-liao, Kaohsiung, TOU-AE 10170, 168 mm TL, TOU-AE 11018, 209 mm TL, TOU-AE 11016, 311 mm TL; **E1, E2**. Collected from Dong-gang, Pingtung, NMMB-P31510, 164 mm TL, TOU-AE 11231, 164 mm TL; **F**. Holotype of *Myrophis heterognathos* Bleeker, 1858, BMNH 1867.11.28.305, 137 mm TL.

##### Coloration.

Preserved specimens, body uniformly brown, with the dorsal surface of the head and the region above the lateral line pores slightly darker. Lateral and ventral surfaces of the head, and the region below the lateral line pores, paler. Lateral line pores pale. Pectoral, vertical fins pale. Peritoneum pale.

##### Size.

Largest specimen examined 275 mm TL.

##### Etymology.

The specific name *nanhaiensis* is derived from “Nan Hai”, the Chinese name for the South China Sea, the region where the holotype was collected, combined with the Latin adjective -*ensis*, meaning “originating in”.

##### Distribution.

Currently known only from the South China Sea at depths of ~ 509–540 m.

##### Remarks.

In the South China Sea region, only *Gnathophis asanoi* and *G. heterognathos* have been recorded. *Gnathophis nanhaiensis* and *G. asanoi* share the absence of elevated lateral-line pores above the pectoral fin, whereas pores are elevated in *G. heterognathos*. *Gnathophis nanhaiensis* differs from *G. asanoi* in having a larger head length (16.9–17.9% vs 13.9–15.0% TL), predorsal length (19.7–20.3% vs 16.0–16.9% TL), preanal length (42.0–43.6% vs 35.2–38.1% TL), trunk length (24.1–26.7% vs 20.2–24.0% TL), upper jaw length (38.3% vs 36.8–37.4% HL), preanal vertebrae (40–42 vs 34–37), precaudal vertebrae (47–48 vs 42–44), preanal pores (37–41 vs 35–37), supraorbital pores (6 vs 5), preopercular pores (3 vs 2), and a shorter tail (56.4–58.0% vs 61.9–64.8% TL).

## Discussion

### Genetic analysis and comparisons

**Figure 9.**
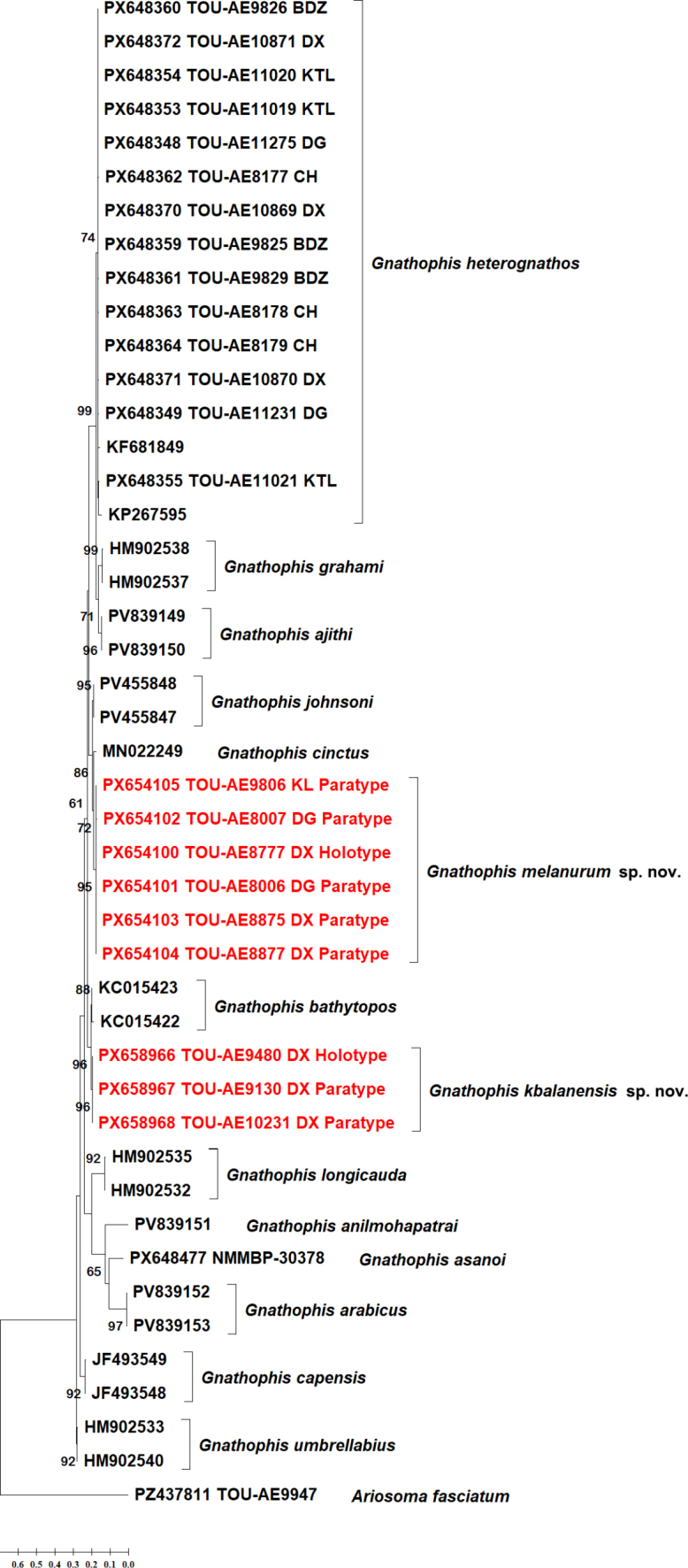
Maximum likelihood tree of *Gnathophis* species reconstructed from COI sequences under the HKY+G substitution model. Bootstrap values are only presented for main branches, and values below 60 are not shown.

Among the currently recognized species of *Gnathophis*, COI sequences representing 12 species were available from public databases and included in the present analyses (Fig. 9): *G. heterognathos* from the western Pacific; *G. grahami* from the southwestern Pacific; *G. johnsoni* from the western and central Pacific; *G. cinctus* from the eastern Pacific; *G. umbrellabius* from the southwestern Pacific; *G. longicauda* from the Indo–West Pacific; *G. asanoi* from the western Pacific; *G. ajithi*, *G. anilmohapatrai*, and *G. arabicus* from the Indian Ocean; *G. bathytopos* Smith & Kanazawa, 1977 from the western Atlantic; and *G. capensis* (Kaup, 1856) from the southeastern Atlantic.

**Table 8.** Distribution of preanal vertebrae of all *Gnathophis* species in Taiwan.

**Taxa**	***n***	**26**	**27**	**28**	**29**	**30**	**31**	**32**	**33**	**34**	**35**	**36**	**37**	**//**	**40**	**41**	**42**
*G. asanoi*	3									1	1	0	1				
*G. heterognathos*	70	4	5	3	1	7	8	12	9	18	2	1					
*G. kbalanensis* sp. nov.	4									2	1	1					
*G. melanurum* sp. nov.	50			1	5	10	20	13									
*G. nanhaiensis* sp. nov.	2														1	0	1

**Table 9.** Distribution of precaudal vertebrae of all *Gnathophis* species in Taiwan.

**Taxa**	***n***	**33**	**34**	**35**	**36**	**37**	**38**	**39**	**40**	**41**	**42**	**43**	**44**	**//**	**47**	**48**
*G. asanoi*	3										1	1	1			
*G. heterognathos*	70	2	5	5	2	12	7	14	13	9	1					
*G. kbalanensis* sp. nov.	4										1	3				
*G. melanurum* sp. nov.	50						8	17	21	3						
*G. nanhaiensis* sp. nov.	2														1	1

**Table 10.** Distribution of total vertebrae of all *Gnathophis* species in Taiwan. Incomplete specimens are not included in the table.

**Taxa**	***n***	**120**	**121**	**122**	**123**	**124**	**125**	**126**	**127**	**//**	**134**	**135**	**136**	**137**	**138**	**139**	**140**	**141**	**142**	**143**
*G. asanoi*	3															1	1	1		
*G. heterognathos*	64	4	6	9	18	9	8	4	6											
*G. kbalanensis* sp. nov.	4										1	1	1	1						
*G. melanurum* sp. nov.	47	2	8	9	11	13	4													
*G. nanhaiensis* sp. nov.	2															1	0	0	0	1

**Table 11.** Distribution of preanal lateral-line pores of all *Gnathophis* species in Taiwan.

**Taxa**	***n***	**22**	**23**	**24**	**25**	**26**	**27**	**28**	**29**	**30**	**31**	**32**	**33**	**34**	**35**	**36**	**37**	**//**	**41**
*G. asanoi*	3														1	1	1		
*G. heterognathos*	70	1	0	0	0	2	8	3	2	5	13	12	10	10	4				
*G. kbalanensis* sp. nov.	4											2	0	1	1				
*G. melanurum* sp. nov.	50								6	14	20	9							
*G. nanhaiensis* sp. nov.	2																1	0	1

**Table 12.** Comparison of *Gnathophis* species with elevated lateral-line pores above the pectoral fin in the Indo-West Pacific. Data sources: A, present study; B, Karmovskaya 2004; C, Karmovskaya and Paxton 2000; D, Prokofiev et al. 2025; E, Ho et al. 2018; F, Matsubara and Ochiai 1951; G, Asano 1962; H, Asano 1958; I, Kodeeswaran and Karmovskaya 2025; J, Kotthaus 1968.

	**LL pores elevated**	**PreA pores**	**PreC vertebrae**	**Total vertebrae**	**POP pores**	**Peritoneum color**	**Distribution**	**Data source**
*G. kbalanensis* sp. nov.	Y	32–35	42–43	134–137	3	black	Northeastern Taiwan	A
*G. melanurum* sp. nov.	Y	29–32	37–41	120–125	3	black	NE and SW Taiwan	A
*G. heterognathos*	Y	22–35	33–42	120–127	3	pale	Japanese to Philippines	A, B, F, G, H
*G. castlei*	Y	31–34	40–45	126–136	2	brown with silver	Eastern Australia	B, C
*G. johnsoni*	Y	31–35	41–43	130–134	3	uniformly dusky	SE-Hawaiian Chain	D
*G. macroporis*	Y	36–40	43–48	125–129	3	dark	Southeastern Australia	C
*G. grahami*	Y	29–33	38–43	125–139	3	dark	Eastern Australia	C
*G. microps*	Y	30–32	39–41	126–128	2	pale	Western Australia	C
*G. nasutus*	Y	25–28	34–37	120–123	2	pale	Western Australia	C
*G. melanocoelus*	Y	28–31	37–39	120–122	3	silver	Western Australia	C
*G. ajithi*	Y	27–33	33–38	122–126	2	silvery	Arabian Sea	I
*G. heterolinea*	Y	28	–	120	2	dark brown	Indian Ocean	I, J

**Table 13.** Comparison of *Gnathophis* species in the Indo-West Pacific region, where the lateral-line pores above the pectoral fin are not elevated. Data sources: A, present study; B, Karmovskaya 2004; C, Karmovskaya and Paxton 2000; D, Prokofiev et al. 2025; E, Ho et al. 2018; F, Matsubara and Ochiai 1951; G, Asano 1962; H, Asano 1958; I, Kodeeswaran and Karmovskaya 2025; J, Sakurai et al. 2014; K, Karrer 1983.

	**LL pores elevated**	**PreA pores**	**PreC vertebrae**	**Total vertebrae**	**POP pores**	**Peritoneum color**	**Distribution**	**Data source**
*G. nanhaiensis* sp. nov.	N	38–40	47–48	139–143	3	pale	South China Sea	A
*G. neocaledoniensis*	N	37–38	45–47	142–145	3	silvery	NE New Caledonia	B
*G. umbrellabius*	N	34–38	41–47	136–149	2	dark	Southeastern Australia	C
*G. asanoi*	N	35–37	42–44	139–141	2	pale	SW Taiwan to NE Philippines	A, B, E
*G. longicauda*	N	30–35	40–43	118–123	3	silver	Southern and Western Australia	C
*G. habenatus*	N	36–38	45–47	122–124	2	dark	New Zealand	C
*G. ginanago*	N	35–40	43–46	126–135	3	pale	Japanese	A, H, J
*G. xenica*	N	41–48	56–58	151–159	2	pale	Japanese	A, F, G, J
*G. anilmohapatrai*	N	36–40	47–48	133–138	2	silvery	Arabian Sea	I
*G. arabicus*	N	27–33	33–38	122–126	2	silvery	Arabian Sea	I
*G. leptosomatus*	N	39	46	156	2	pale	Indian Ocean	I, K

The pairwise genetic distance analysis with the K2P model interspecific distance between *G. kbalanensis* sp. nov. and *G. melanurum* sp. nov. and the other twelve congeneric species (Table 2).

*Gnathophis kbalanensis* sp. nov. exhibited pairwise COI genetic distances from its congeners as follows: 5.7–5.9% from *G. grahami*, 4.7–5.7% from *G. heterognathos*, 4.0–4.2% from *G. johnsoni*, 5.3% from *G. cinctus*, 6.1–6.3% from *G. umbrellabius*, 8.5–9.2% from *G. longicauda*, 12.7% from *G. asanoi*, 5.1–5.5% from *G. ajithi*, 13.7% from *G. anilmohapatrai*, 12.9–13.2% from *G. arabicus*, 1.0–1.8% from *G. bathytopos*, and 5.3–5.5% from *G. capensis*.

Similarly, *G. melanurum* sp. nov. shows clear genetic separation from other species, with pairwise distances of 5.9–6.6% from *G. grahami*, 5.1–6.6% from *G. heterognathos*, 2.0–2.6% from *G. johnsoni*, 2.0–2.4% from *G. cinctus*, 7.4–8.5% from *G. umbrellabius*, 7.6–9.0% from *G. longicauda*, 12.5–12.7% from *G. asanoi*, 5.9–6.8% from *G. ajithi*, 12.5–12.9% from *G. anilmohapatrai*, 12.0–12.9% from *G. arabicus*, 4.7–5.9% from *G. bathytopos*, and 6.8–7.9% from *G. capensis*. In addition, *G. kbalanensis* sp. nov. and *G. melanurum* sp. nov. differ from each other by 4.4–4.9% in pairwise COI distance, further supporting their recognition as distinct species.

Specimens of *G. heterognathos* collected from waters off Taiwan show intraspecific genetic divergence (Fig. 9), with maximum pairwise distances reaching 1.4% (PX648355). Comparisons with sequences identified as *G. nystromi* retrieved from NCBI database (KF681849, KP267595) reveal genetic distances of up to 1.8% (KP267595). After comparing the morphological characters of specimens of *G. heterognathos* collected from Taiwanese waters with those of *G. nystromi* collected from Japanese waters, the data were found to overlap extensively, and no differences were observed in dentition characters. Therefore, there is currently no evidence supporting their recognition as two separate valid species. The observed genetic distance of up to 1.8% is presumed to be related to their wide geographic distribution. These values fall within the range of intraspecific variation observed in the present dataset. Further studies will continue to investigate whether *G. heterognathos* and *G. nystromi* collected from Taiwanese and Japanese waters, respectively, represent valid distinct species.

When compared with closely related species, *G. heterognathos* exhibits pairwise genetic distances of 3.0–3.6% from *G. grahami* and 2.4–3.0% from *G. ajithi*. Based on these comparisons, the maximum intraspecific COI divergence in *G. heterognathos* is inferred to be 1.8%, supporting the conspecific status of Taiwanese material and previously published sequences.

### Comparison of *Gnathophis kbalanensis* sp. nov. with Indo-Pacific congeners

*Gnathophis kbalanensis* sp. nov. belongs to the Indo-Pacific assemblage of *Gnathophis* species characterized by elevated lateral line pores above the pectoral fin, a condition shared with *G. castlei*, *G. johnsoni*, *G. macroporis*, *G. grahami*, *G. microps*, *G. nasutus*, *G. melanocoelus*, *G. heterolinea*, and *G. ajithi*. Within this group, *G. kbalanensis* sp. nov. can be clearly distinguished from all congeners by a unique combination of vertebral counts, proportional measurements, sensory-pore patterns, and coloration.

Compared with *G. microps*, *G. melanocoelus*, *G. ajithi*, *G. kbalanensis* sp. nov. has more total vertebral counts (134–137 vs 126–128 in *G. microps*, 120–122 in *G. melanocoelus*, 122–126 in *G. ajithi*), precaudal vertebrae (42–43 vs 39–41 in *G. microps*, 37–39 in *G. melanocoelus*, 33–38 in *G. ajithi*), preanal pores (32–35 vs 30–32 in *G. microps*, 28–31 in *G. melanocoelus*, 27–33 in *G. ajithi*), preopercular pores (3 vs 2 in *G. microps*, *G. ajithi*, *G. heterolinea*), a shorter head length (16.4–17.1% in TL vs 17.4–18.8% in TL in *G. microps*, 18.1–19.1% in TL in *G. melanocoelus*, 18.0–19.1% in TL in *G. ajithi*), and color of peritoneum (black vs pale in *G. microps*, silver in *G. melanocoelus*, *G. ajithi*). *Gnathophis kbalanensis* sp. nov. differs from *G. castlei*, *G. johnsoni*, *G. macroporis*, *G. heterolinea* in having more total vertebrae (134–137 vs 126–136 in *G. castlei*, 130–134 in *G. johnsoni*, 125–129 in *G. macroporis*, 120 in *G. heterolinea*), fewer precaudal vertebrae (42–43 vs 43–48 in *G. macroporis*), preanal pores (32–35 vs 36–40 in *G. macroporis*), a shorter head length (16.4–17.1% in TL vs 17.2–20.0% in TL in *G. johnsoni*), trunk length (21.0–22.8% in TL vs 23.4–25.3% in TL in *G. macroporis*), a longer head length (16.4–17.1% in TL vs 13.9–16.4% in TL in *G. macroporis*), trunk length (21.0–22.8% in TL vs 18.2–21.0% in TL in *G. castlei*), preanal length (38.3–39.7% in TL vs 35.5–38.7% in TL in *G. castlei*). The eye diameter of *G. kbalanensis* sp. nov. is less than *G. grahami* (17.2–17.8% in HL vs 20.2–26.9% in HL). Finally, *G. kbalanensis* sp. nov. differs from *G. nasutus* in having more precaudal vertebrae (42–43 vs 34–37), preanal pores (32–35 vs 25–28), a longer trunk length (21.0–22.8% in TL vs 15.1–18.8% in TL), preanal length (38.3–39.7% in TL vs 30.8–38.1% in TL), and smaller eye diameter (17.2–17.8% in HL vs 18.6–27.1% in HL).

Overall, the combination of elevated lateral line pores, high vertebral counts, distinctive proportional measurements, and color of peritoneum uniquely diagnoses *G. kbalanensis* sp. nov. among Indo-Pacific congeners. These morphological distinctions are congruent with the observed COI genetic divergence and support the recognition of *G. kbalanensis* sp. nov. as a distinct species within the genus.

### Comparison of *Gnathophis melanurum* sp. nov. with Indo-Pacific congeners

*Gnathophis melanurum* sp. nov. belongs to the Indo-Pacific assemblage of *Gnathophis* species characterized by elevated lateral line pores above the pectoral fin, a condition shared with *G. castlei*, *G. johnsoni*, *G. macroporis*, *G. grahami*, *G. microps*, *G. nasutus*, *G. melanocoelus*, *G. heterolinea*, and *G. ajithi*. Within this group, *G. melanurum* sp. nov. is readily distinguished from all congeners by a consistent combination of vertebral counts, sensory-pore patterns, body proportions, and especially the distinctive coloration of the vertical fins.

Compared with *G. castlei*, *G. johnsoni*, *G. macroporis*, *G. melanurum* sp. nov. has fewer preanal pores (29–32 vs 31–34 in *G. castlei*, 31–35 in *G. johnsoni*, 36–40 in *G. macroporis*), precaudal vertebrae (37–41 vs 40–45 in *G. castlei*, 41–43 in *G. johnsoni*, 43–48 in *G. macroporis*), total vertebrae (120–125 vs 126–136 in *G. castlei*, 130–134 in *G. johnsoni*, 125–129 in *G. macroporis*). And the new species has fewer total vertebrae than *G. grahami* and *G. microps* (120–125 vs 125–139 in *G. grahami*, 126–128 in *G. microps*). From *G. melanocoelus*, *G. ajithi*, *G. melanurum* sp. nov. differs in having a shorter head length (16.1–18.7% in TL vs 18.1–19.1% in TL in *G. melanocoelus*, 18.0–19.1% in TL in *G. ajithi*), color of peritoneum (black vs silver in *G. melanocoelus*, silver in *G. ajithi*). In comparison with *G. nasutus*, *G. heterolinea*, *G. melanurum* sp. nov. shows longer trunk length (17.8–23.0% in TL vs 15.1–18.8% in TL), more precaudal vertebrae (37–41 vs 34–37), preanal pores (29–32 vs 25–28), preopercular pores (3 vs 2), and color of peritoneum (black vs pale).

Finally, *G. melanurum* sp. nov. can be distinguished from other eight species in having color of vertical fins (dorsal and anal fins pale, caudal fin black). *G. castlei*, *G. johnsoni*, *G. macroporis*, *G. grahami*, *G. microps*, *G. nasutus* color of vertical fins are pale with black margin. *G. melanocoelus* color of vertical fins are pale. *G. ajithi* color of dorsal and anal fins pale with black margin, caudal fin dark and tip white.

Overall, *G. melanurum* sp. nov. is well diagnosed by its combination of fewer vertebral counts relative to most Indo-Pacific congeners, a higher number of preopercular pores, distinctive proportional characters, and a unique vertical fin coloration pattern (pale dorsal and anal fins with a black caudal fin). These morphological differences are congruent with the observed COI genetic divergence and support the recognition of *G. melanurum* sp. nov. as a distinct species within the genus.

### Comparison of *Gnathophis nanhaiensis* sp. nov. with Indo-Pacific congeners

*Gnathophis nanhaiensis* sp. nov. belongs to the Indo-Pacific assemblage of *Gnathophis* species characterized by lateral line pores above the pectoral fin not elevated, a condition shared with *G. asanoi*, *G. ginanago*, *G. xenica*, *G. neocaledoniensis*, *G. longicauda*, *G. umbrellabius*, *G. habenatus*, *G. leptosomatus*, *G. anilmohapatrai*, and *G. arabicus*. Within this group, *G. nanhaiensis* sp. nov. can be distinguished from all congeners by a consistent combination of high vertebral counts, greater proportional measurements of the head, trunk, and preanal region, and a suite of sensory-pore characters.

Compared with *G. ginanago*, *G. longicauda*, *G. habenatus*, *G. anilmohapatrai*, *G. arabicus*, *G. nanhaiensis* sp. nov. has higher total vertebrae (139–143 vs 126–135 in *G. ginanago*, 118–123 in *G. longicauda*, 122–124 in *G. habenatus*, 133–138 in *G. anilmohapatrai*, 122–126 in *G. arabicus*), precaudal vertebrae (47–48 vs 43–46 in *G. ginanago*, 40–43 in *G. longicauda*, 45–47 in *G. habenatus*, 33–38 in *G. arabicus*), preanal pores (37–41 vs 30–35 in *G. longicauda*, 36–38 in *G. habenatus*, 27–33 in *G. arabicus*), more POP pores (3 vs 2 in *G. habenatus*, *G. anilmohapatrai*, *G. arabicus*), a longer predorsal length (19.7–20.3% in TL vs 18.0–18.8% in TL in *G. habenatus*, 17.4–19.6% in TL in *G. anilmohapatrai*, 16.5–19.4% in TL in *G. arabicus*), preanal length (42.0–43.6% in TL vs 36.9–40.7% in TL in *G. longicauda*, 38.7–42.2% in TL in *G. anilmohapatrai*, 33.7–39.0% in TL in *G. arabicus*), trunk length (24.1–26.7% in TL vs 20.5–23.2% in TL in *G. longicauda*, 19.7–24.1% in TL in *G. arabicus*), the color of peritoneum (pale vs silver in *G. ginanago*, *G. longicauda*, *G. anilmohapatrai*, *G. arabicus*, dark in *G. habenatus*). From *G. leptosomatus* and *G. nanhaiensis* sp. nov. differs in having a fewer total vertebrae (139–143 vs 156 in *G. leptosomatus*). Finally, *G. nanhaiensis* sp. nov. differs from *G. xenica*, *G. neocaledoniensis*, *G. umbrellabius* in having more precuadal vertebrae (47–48 vs 45–47 in *G. neocaledoniensis*, 41–47 in *G. umbrellabius*), fewer precaudal vertebrae (47–48 vs 56–58 in *G. xenica*), a longer head length (16.9–17.9% in TL vs 13.6–14.2% in TL in *G. xenica*), predorsal length (19.7–20.3% in TL vs 15.7–15.8% in TL in *G. xenica*, 16.4–18.9% in TL in *G. umbrellabius*), trunk length (24.1–26.7% in TL vs 19.4–21.6% in TL in *G. neocaledoniensis*, 20.4–23.9% in TL in *G. umbrellabius*), preanal length (42.0–43.6% in TL vs 39.0–39.1% in TL in *G. xenica*, 38.4–40.0% in TL in *G. neocaledoniensis*, 36.1–40.4% in TL in *G. umbrellabius*), a larger eye diameter (17.8–19.4% in HL vs 16.7–17.9% in HL in *G. neocaledoniensis*), a shorter head length (16.9–17.9% in TL vs 18.3–18.9% in TL in *G. neocaledoniensis*), predorsal length (19.7–20.3% in TL vs 20.8–21.9% in TL in *G. neocaledoniensis*), trunk length (24.1–26.7% in TL vs 27% in TL in *G. xenica*), a smaller eye diameter (17.8–19.4% in TL vs 22.2–25.5% in HL in *G. xenica*, 19.2–27.7% in HL in *G. umbrellabius*).

Overall, *G. nanhaiensis* sp. nov. is distinguished from Indo-Pacific congeners with non-elevated lateral line pores by its combination of high vertebral counts, elongate trunk and preanal region, distinct cranial proportions, and sensory-pore configuration. These morphological distinctions are congruent with the observed COI genetic divergence and support the recognition of *G. nanhaiensis* sp. nov. as a distinct species within the genus.

### Comparative materials

***Gnathophis xenica***: NMMM-P34730 (formerly HUMZ 229180) (1, 215 mm TL), 37°35'31"N–37°36'82"N, 141°33'18"E–141°33'92"E, off northeastern Japan, bottom trawl, 01 Nov. 2017. ***Gnathophis ginanago***: NSMT-P78226 (3, 198–392 mm TL, 81.5–160 mm PAL), off Sagami Bay, Kanagawa. NSMT-P81277 (1, 338 mm TL, 135 mm PAL), off Sagami Bay, Kanagawa. HUMZ 208625 (1, 277 mm TL, 105 mm PAL), off Misawa, Aomori, Japan. HUMZ 56949 (1, 259 mm TL, 102 mm PAL), Onhama Fish Market, Iwaki, Fukushima, Japan. HUMZ 206331 (1, 303 mm TL, 125 mm PAL), off Misawa, Aomori, Japan. HUMZ 212716 (1, 343 mm TL, 145 mm PAL), off Misawa, Aomori, Japan. HUMZ 212717 (1, 313 mm TL, 130 mm PAL), off Misawa, Aomori, Japan. ***Gnathophis nystromi***: KPM-NI 62228 (1, 296 mm TL, 110 mm PAL), off Miyazaki. KPM-NI 66034 (1, 250 mm TL, 100 mm PAL), off Fukushima. KPM-NI 2463 (1, 270 mm TL, 105 mm PAL), off Sagami Bay, Kanagawa. KPM-NI 67008 (1, 373 mm TL, 150 mm PAL), off Miyagi. ***Gnathophis heterognathos* from Japan**: NSMT-P93607 (1, 300 mm TL, 117 mm PAL), off Kochi. NSMT-P100075 (1, 293 mm TL, 111 mm PAL), off Kochi.

## Supplementary Material

XML Treatment for Gnathophis

XML Treatment for Gnathophis asanoi

XML Treatment for Gnathophis heterognathos

XML Treatment for Gnathophis kbalanensis

XML Treatment for Gnathophis melanurum

XML Treatment for Gnathophis nanhaiensis

## Additional information

### Artificial Intelligence (AI) use

The authors accept full responsibility for the content of the manuscript, including the disclosure of any use of AI.

No AI tools were used in the preparation of this manuscript.

### Author ORCIDs

J.-F. Huang https://orcid.org/0000-0001-5213-886X

Y.-C. Liao https://orcid.org/0000-0002-9158-1159

T.-Y. Chan https://orcid.org/0000-0002-8143-0007

H.-M. Chen https://orcid.org/0000-0002-3921-2022

### Data availability

All of the data that support the findings of this study are available in the main text.
